# Deep Learning Intervention for Health Care Challenges: Some Biomedical Domain Considerations

**DOI:** 10.2196/11966

**Published:** 2019-08-02

**Authors:** Igbe Tobore, Jingzhen Li, Liu Yuhang, Yousef Al-Handarish, Abhishek Kandwal, Zedong Nie, Lei Wang

**Affiliations:** 1 Center for Medical Robotics and Minimally Invasive Surgical Devices Shenzhen Institutes of Advance Technology Chinese Academy of Sciences Shenzhen China; 2 Graduate University Chinese Academy of Sciences Beijing China

**Keywords:** machine learning, deep learning, big data, mHealth, medical imaging, electronic health record, biologicals, biomedical, ECG, EEG, artificial intelligence

## Abstract

The use of deep learning (DL) for the analysis and diagnosis of biomedical and health care problems has received unprecedented attention in the last decade. The technique has recorded a number of achievements for unearthing meaningful features and accomplishing tasks that were hitherto difficult to solve by other methods and human experts. Currently, biological and medical devices, treatment, and applications are capable of generating large volumes of data in the form of images, sounds, text, graphs, and signals creating the concept of big data. The innovation of DL is a developing trend in the wake of big data for data representation and analysis. DL is a type of machine learning algorithm that has deeper (or more) hidden layers of similar function cascaded into the network and has the capability to make meaning from medical big data. Current transformation drivers to achieve personalized health care delivery will be possible with the use of mobile health (mHealth). DL can provide the analysis for the deluge of data generated from mHealth apps. This paper reviews the fundamentals of DL methods and presents a general view of the trends in DL by capturing literature from PubMed and the Institute of Electrical and Electronics Engineers database publications that implement different variants of DL. We highlight the implementation of DL in health care, which we categorize into biological system, electronic health record, medical image, and physiological signals. In addition, we discuss some inherent challenges of DL affecting biomedical and health domain, as well as prospective research directions that focus on improving health management by promoting the application of physiological signals and modern internet technology.

## Introduction

The continuous advancement in medicine, genome, pharmaceutical, and health care monitoring is a result of the development and application of technological devices. This has made it possible to easily capture data for analysis and processing. Similarly, improvement in technology also makes it possible to store very large amount of data with useful information. Currently, camera to detect the movements of monitored patients (Panasonic BL-C230A), wireless necklace and badges for acquisition of bioacoustic signals and blood flow, wearable fiber-type smart material, cuffless blood pressure meter, and sensor devices are capable of generating large volumes of data in the form of images, sounds, text, graphs, and signals creating the concept of big data [1-4]. The term big data can be described as the exponential growth and wide availability of discrete, continuous, categorical, or hybrid data, which are difficult or even impossible to manage and analyze using conventional software tools and technologies [5,6]. Furthermore, estimate shows that 30% of the world storage was occupied by medical images in 2011 and will progressively increase in subsequent years [2,7]. This shows the extremely large and often underestimated amount of data produced in medical institutions. Mobile health (mHealth) is referred to as one of the technological breakthroughs in this decade [8]. The global proliferation of mobile devices and health applications has made mHealth synonymous with big data. This large amount of unutilized generated data calls for attention.

**Figure 1:**
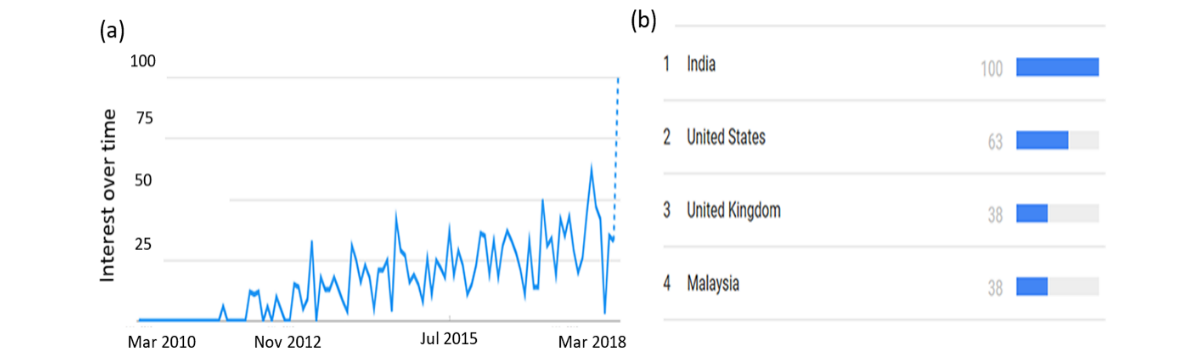
Google Trends for “big data in healthcare” between 2010 and 2018; (a) occurrence timeline graph and (b) prevalence occurrence by country.

Big data provides the opportunity for health policy experts, physicians, and health care institutions to make data-driven judgments that will enhance patient treatment, disease management, and health care decisions. Many experts have used internet tools for big data services and related applications. This is depicted in the graph in Figure 1, which was obtained from Google Trends for “big data in healthcare” between 2010 and 2018. Google Trends is a free Web service by Google Inc that provides statistical occurrence of activities by people on the internet all over the world. The trend in the graph is calculated as interest over time on a scale from 0 to 100, where 100 refers to the maximum computed score for total search and related activity for the topic.

**Figure 2:**
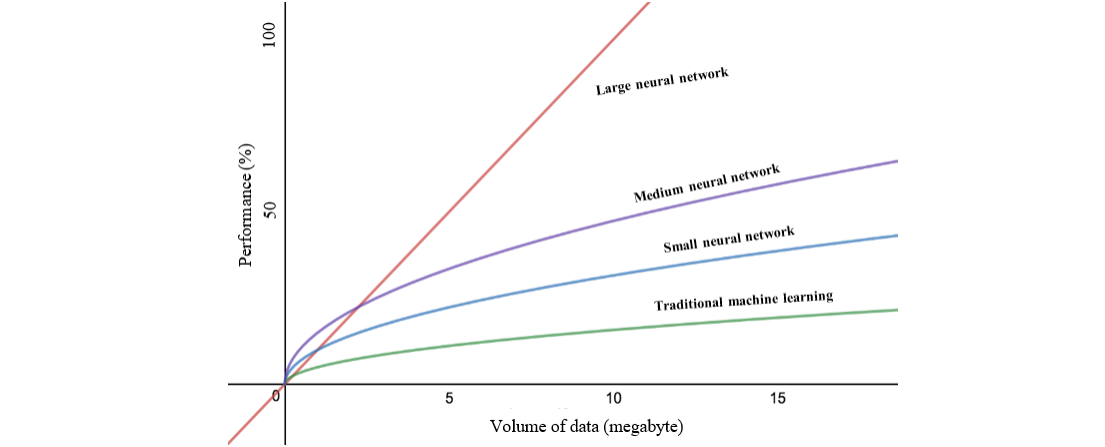
How machine learning techniques scale with amount of data.

The first graph in Figure 1 shows the continuous rise in activities regarding big data in health care, and the top 5 countries where it was most popular is given in the second graph, with India, United States, and United Kingdom leading the occurrence chat. The size of medical data is too large for comprehensive analysis with the available analytical tools to maximize the knowledge available in big data. Traditional machine learning (ML) techniques and algorithms have limited capacity to utilize big data and, in most cases, the solution becomes complex and undesirable. Deep learning (DL) is proposed and provides a prospective solution to this challenge. Figure 2 shows the performance between DL and other ML techniques in the situation of increasing data size. The primary advantage of DL is that the performance of large architecture of DL increases with increase size of available data [9].

**Figure 3:**
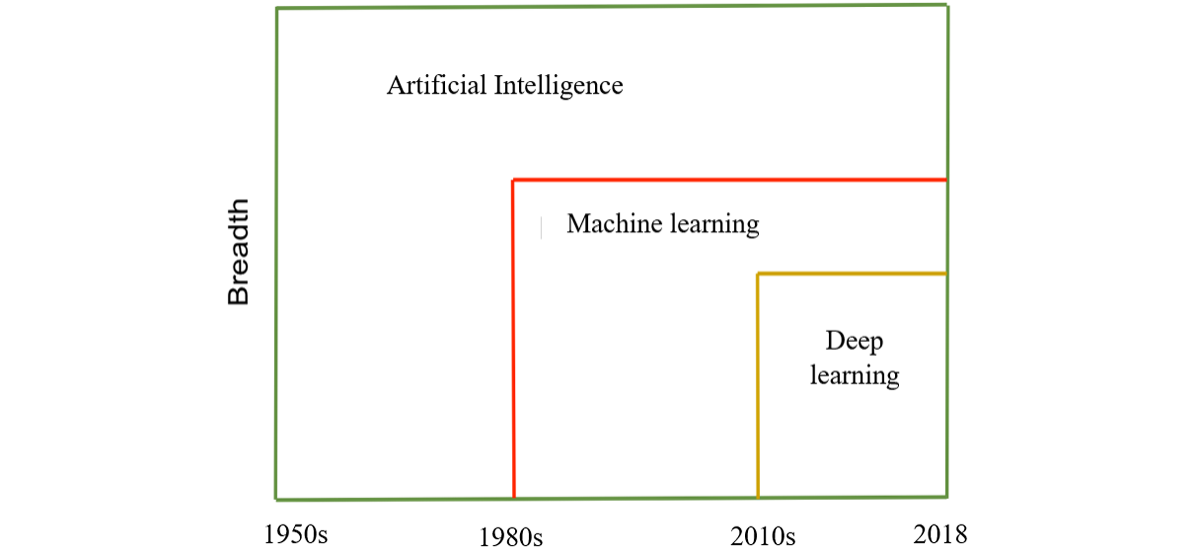
Relationship between artificial intelligence, machine learning and deep learning with emerging timeline.

The main question will be what is DL? Human experts in a specific domain have ample knowledge about the subject in that domain. The limitation with human experts is because of their subjectivity, large variations across interpreters, availability, and fatigue [10,11]. To help the accomplish task performed by humans and overcome these limitations, intelligence demonstrated by humans is built into machines and computers to create the concept of artificial intelligence (AI). ML is a branch of AI that gives computers the ability to learn and perform the role of experts without being explicitly programmed [12,13]. Some examples of ML include support vector machine (SVM), decision tree, logistic regression, Naïve Bayes, K-means clustering, and so on. On the basis of a broad classification scheme, ML can be categorized into 3 groups [13-15]. The first is supervised learning; the computer learns the classification system from the class labels provided. The second is unsupervised learning, where no labels are given; the purpose is to program the computer to do things without telling it how to do it. The third is semisupervised, where the computer learns from a combination of available and unavailable labeled data; usually the size of unavailable labeled data for learning is larger. The recent hunger for data consumption and analysis has opened up new frontier for more ideas and applications [16]. Artificial neural network or neural network (NN) is another example of ML, with an interconnection of nodes called neurons with 3 major layers, input, hidden, and output layers, where the hidden layer is a single layer that connects the input layer to the output layer. The purpose of NN is to gradually approximate a function that maps an input to a corresponding output through an iterative optimization process. NN has transformed from its inception as a simple perceptron to solve simple problem to the advanced concept of deep neural network (DNN), which has many cascaded interconnected hidden layers that are able to process and analyze audios, text, signals, images, and more complex data types. DL is the recurrent learning process performed in DNN that enables it to find an optimal function for representing data. The innovation of DL is a developing trend in data analysis and is ranked as one of the best inventions in technologies [17]. DNN is an active branch of ML and its goal is to make machines think and understand as humans by mimicking the grid of the human brain connection, to focus on learning data representation (DR) rather than task-specific algorithms [14].  Figure 3 reveals the relationship between DL, ML, and AI. Currently, DL is set to take over the ML space because of its increasing attention and performance.

**Figure 4:**
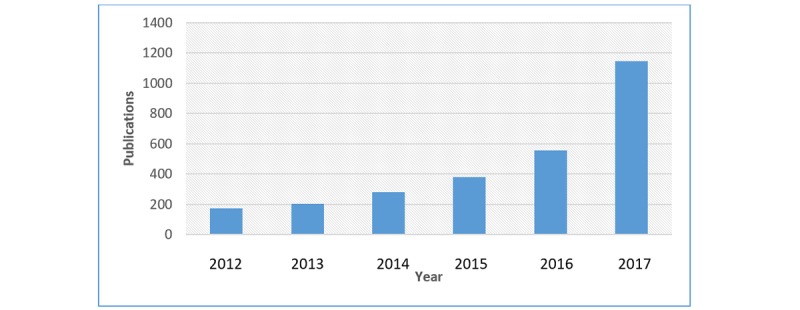
Trends of published papers that implement deep learning techniques. The data are generated by searching for “deep learning” on PubMed database.

ML and DL have in recent times attracted a lot of awareness from different sectors such as academia, industry, media, security, and government alike, and its impact on biomedical and health care cannot be over emphasized. DNN has been applied to solve many traditional problems where available large data need to be analyzed and many impressive results have been reported in different areas such as medical image processing [18], speech analysis [19], and electronic health record (EHR) translation [20,21]. Figure 4 shows the trend in application of DL found in research publications. The number in 2017 is almost twice that of 2016. The trend observed in the figure is a result of the performance and results achieved by DL. Therefore, we can say that more areas and domains are moving toward DL to achieve high performance and better results.

Currently, DL has started making huge impact across different areas in health care. The increasing availability of health care data and rapid development of variations in DL techniques have made it possible to have the impressive results recorded in health care [22,23]. DL techniques can reveal clinically relevant information hidden in large amount of health care data, which in turn can be used for decision making, treatment, control, and prevention of health conditions. Some application areas of DL include health behavior reaction [24,25], EHR processing and retrieving scientifically sound treatment from text [26,27], eye related analysis and classification [28-30], gait analysis and robotic-assisted recovery [31,32], hearing disorder treatment [33], cancer treatment [34,35], heart diagnosis [36,37], and brain activity analysis [38-40]. This makes the treatment easier for health care provider and convenient for patients, with faster and productive monitoring. The advancement in DL in medicine has translated the use of simple equipments, such as thermometer and stethoscope, into computed tomography (CT), ultrasound diagnostic devices, radio nuclear imaging, radiation therapy, lithotripsy, dialysis, ventilators, and so on, which have taken conventional patient care to highly adaptive treatment, capable of challenging many dreaded diseases [23]. There is no doubt that in the coming years, health care treatment and equipment will witness greater improvements in many more areas, to make it more effective with qualitative services.

Compared with the traditional ML algorithm, the depth of learning and feature extraction in DL has unparalleled superiority. The deep network structure can realize the approximation of complex functions through nonlinear transformation in the hidden layers. From low to high level, the representation of features is more and more abstract, and the original data can be characterized more accurately [41]. A large number of experimental works have applied DL models and techniques and there are variants of DL models. The goal of this paper is not to show all the techniques and models, but to highlight the important principles and the applications of DL in health care and medical field. A simple feedforward DNN architecture is the autoencoder (AE) that comprises encoder and decoder functions for input and output layers, respectively. Convolutional neural networks (CNNs) have had the greatest impact within the field of health informatics [42]. Its architecture can be described as an interleaved set of feedforward layers implementing convolutional filters followed by reduction, rectification, or pooling layers. For each layer, the CNN creates high-level abstract feature. Another variant of DL is the recurrent neural network (RNN), which is a sequential data NN with an inbuilt memory that updates the state of each neuron with previous input. The deep belief network (DBN) model has only several layers of hidden units and there is connection between each unit in a layer with each unit in the next layer. Another architecture is the deep Boltzmann machine (DBM) which has completely undirected connections, unlike DBN, between neurons in all layers. DL is computationally intensive. The success and proliferation recorded in DL can be attributed to the advancement in graphics processing units (GPUs), which play a significant role in accelerating the computation requirement of DL [43,44].

The proceeding sections of this paper are organized as follows. We discuss 5 common DL techniques and their basic principle of operation in the next section that describes DL methods. A review of literature in health care and biomedical domains that have applied DL was examined and presented in the section review of DL implementation in health care. In the section challenges in health care for DL applications, we discuss challenges and setbacks encountered in the application of DL and plausible solutions. In the section future trends for deep learning, we present critical discussion about the future trends for DL algorithm for health and biomedical field, and the conclusion section of the paper closes the discussion.

## Deep Learning Methods

### Basic Principles of Operation

In this section, we describe the principles of operation of 5 DL models. There are several variants for each model. The underlying principle is to approximate a function that produces the expected output for a given input. The different models are more suited to handle different challenges and for different kinds of data type and expected task to be performed. The model more suited for image classification is different from speech or time series classification. Some models can be applied as a preprocessing phase to reduce the dimensionality of the data. Basically, the structure of the model comprises interconnected neurons, connecting the input to the output, known as the hidden layer. Therefore, this produces a sequence of activation through the weighted connection from neurons perceiving the environment (input); this is referred to as feedforward [45]. The differences between DL model and NN include the use of more hidden layers in DL compared with NN, which only has 1 or 2 hidden layers. DL can be trained for both unsupervised and supervised learning tasks but NN can only be trained for supervised learning task. At the end of the feedforward process, the result from the output unit is evaluated with the expected value. This evaluation will produce an error value that will lead to the adjustment of connected weight working backward from the output layer to the hidden layer and to the input layer until the output is close to the expected result. This procedure is referred to as backpropagation [46,47].

### Autoencoders

**Figure 5:**
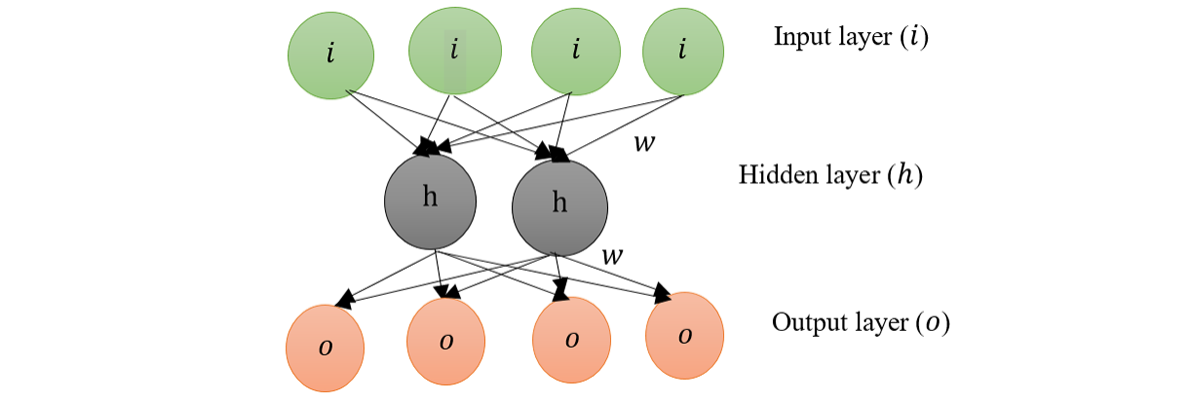
Structure of a simple autoencoder showing input, hidden, and output layers. The interconnection between the neurons is shown in the direction of the arrows.

AE is designed for feature extraction using data-driven learning. It is trained in an unsupervised manner as it is trained to recreate the input vector rather than assign class label. The normal design and structure of AE is to have the same number of neurons in the output and input layers, with full connections between neurons in each layer to subsequent layer as shown in Figure 5. The number of neurons in the hidden layer is smaller than the input and output layer. The purpose of this structure is to encode data in low dimensionality space and to achieve extraction of features. However, where the dimensionality of the data is high to achieve the same purpose, many AE can be stacked together to create a deep AE architecture. There are many deviations of AE presented over the last decade to handle different data patterns for performing specific functions. For example, denoising AE was first proposed by Vincent et al [48]. The purpose was to increase the robustness of the regular AE model. The method recreates the input introducing some noise to the patterns, thus forcing the model to capture just the structure of the input. Another variation is the sparse AE that forces the representation to be sparse, which is used to make the data more separable [49]. Another idea proposed shared weights between nodes to preserve spatial locality and process 2-dimensional (2D) patterns called the convolutional AE [50]. Contractive AE is similar to denoising AE, but instead of injecting noise to corrupt the training set, it modifies the error function by adding analytic contractive cost [51,52]. The learning process for AE is described as minimizing a loss function *L* such that *L(i, g (f (i)))*. *f(i)* is a function that maps *i* to *h* and the function *g* maps *h* to the output which is a reconstruction of the input. *w* is the weight connecting the layers.

### Recurrent Neural Network

This class of DL has connections between neurons in the hidden layer to form a sequence of directed graph. This feature gives it a temporal dynamic state. This is important in applications where the output depends on the previous computations such as the analysis of text, sounds, DNA sequences, and continuous electric signals from the body. The training of RNN is performed with data that have interdependencies to maintain information about what occurred in the previous interval. The performance result at time *t-1* affects the choice at time *t*. It considers the previous output (*O*_t-__1_) and current input (*I*_t_) and produces a number between 0 and 1 from the cell state *M*_t-__1_, where 1 represents *save this value* and 0 represents *dispose this value*. This decision is made by a sigmoid layer called the *gate layer*.

Therefore, the principle of RNN is to define recurrent relation over time steps which can be approximated with the formula: *M*_k_= *f(M*_k-1_ *× W*_r_ *× I*_k_ *× W*_i_*)*, where *M*_k_ is the state at time *k*, *M*_k-1_ is the output of the previous state, *I*_k_ is the input at time *k*, and *W*_r_ and *W*_i_ are the weight parameters in the network. As a result, RNN can be viewed as a state with feedback loop. The final output of the network *O* at a certain time step *k* is typically computed from one or more states such as *M*_k-1_ *… M*_k+j_ and *j=1,2, …k-1*.

**Figure 6:**
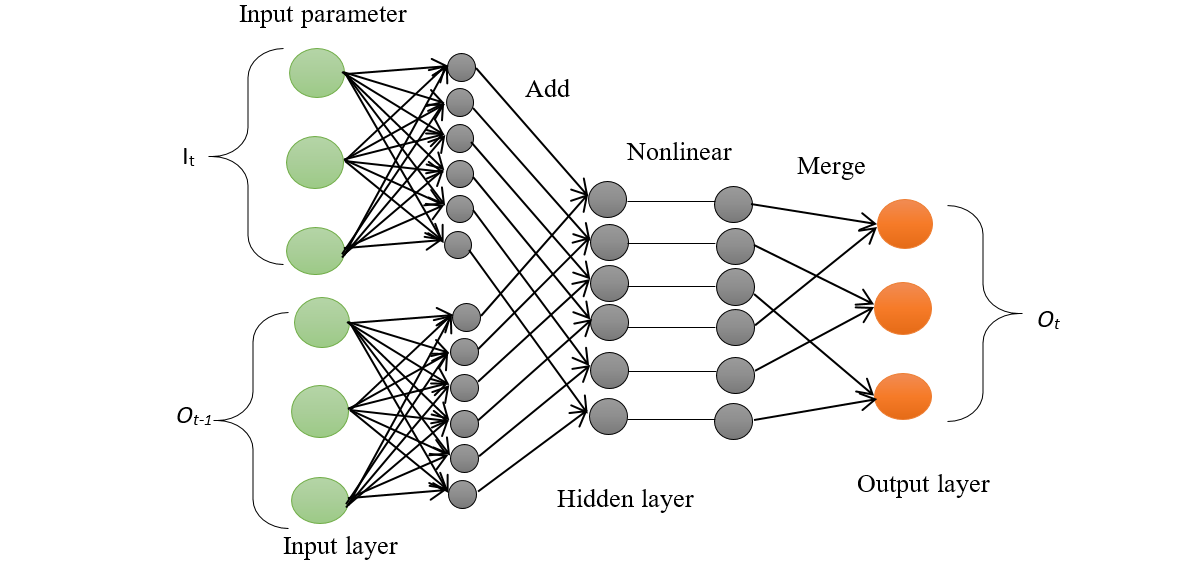
Feedforward recurrent neural network implementation. The final output from the output layer is fed back as part the input in the input layer. Where It and Ot are the input and output at time t and Ot-1 is the output for the previous input at time t-1.

**Figure 7:**
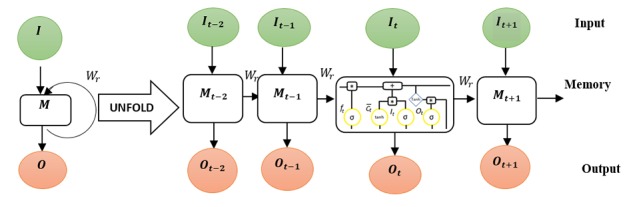
Long short-term memory representation for output sequence influence by input sequence and previous output. Where It-2 and It-1, Ot-2 and Ot-1, Mt-2 and Mt-1 are inputs, outputs, memory respectively for previous time steps. It, Ot, and Mt are the current input, output and memory state of the LSTM cell. Ot+1, and Mt+1 represent subsequent output and memory state respectively for subsequent time step input It+1. I, O, M represent the recurrent input, output and memory state respectively for a simplified LSTM cell operation and Wr is the weight for the computation in the cell.

Hence, the result of new data is dependent on 2 sources of input, the present and the recent past. Owing to this principle, RNNs are said to operate with memory [53]. Figure 6 shows a sample of RNN structure and the connection between neurons in each layer. Beside the structural difference, RNN uses the same weight across for all layers, but other DL uses different weights. This significantly cuts down the total number of parameters that the network needs to learn. Despite the successful application of this model, the setback includes vanishing gradient by long input sequence and exploding gradient problems as described in [54]. To handle the limitation, long short-term memory unit (LSTM) was invented by [55]. Specifically, LSTM in Figure 7 is particularly suitable for applications where there are very long time lags of unknown sizes between important events.

To achieve this, LSTMs utilize new sources of information so that data can be stored in, written to, or read from a node at each step. During the training, the network learns what to store and when to allow either reading or writing so as to minimize the classification errors [56]. Another variant of RNN is the gated recurrent unit, which is a simplified model of LSTM with an equal performance as LSTM [57].

### Convolutional Neural Network

**Figure 8:**
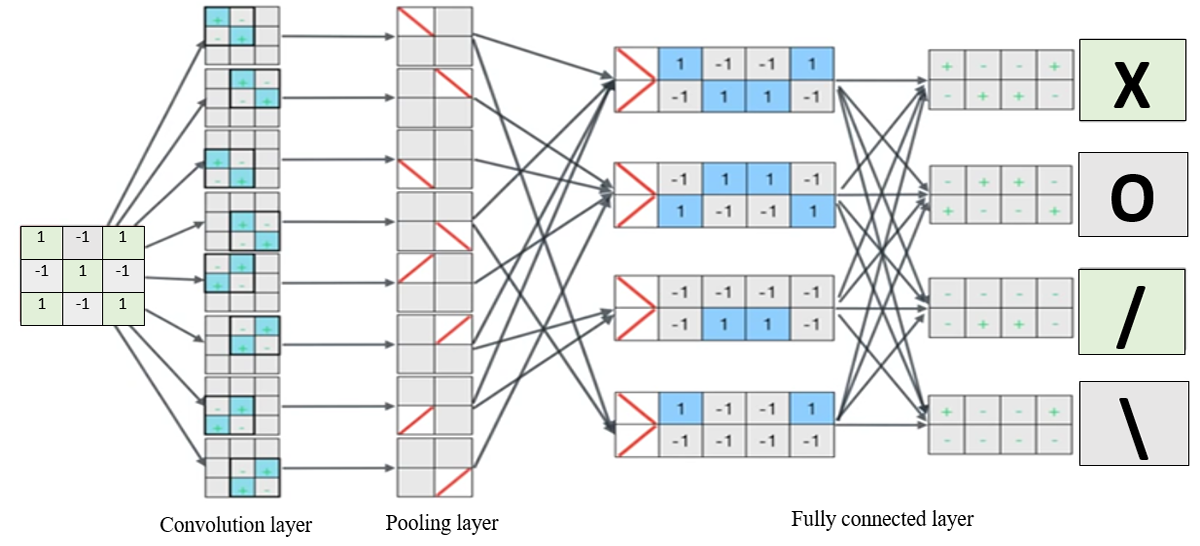
Simple implementation of convolutional neural network to show the sequence of operation to identify “X” with 2 filters.

CNN was inspired by biological processes of the human brain, where the connectivity pattern between neurons resembles the concept of the human visual cortex [58,59]. A typical CNN comprises an input, multiple hidden layers, and an output layer. The hidden layers of a CNN usually comprise the following constituents: convolutional, pooling, fully connected (FC), and normalization layers. An example of CNN was proposed to analyze imagery data [60]. Figure 8 shows a simple implementation to identify a character from a 3×3 pixelated matrix image sliding 2 filters of size 2×2 square matrix (kernel=2) with stride of 1. The example is designed to recognize *X*, O, **,** and */* characters. The convolution layer applies the filter across the input image. The operation is performed with 2 filters over the input image and having the same weight. This produces a total of 8 parameters. In this example, the bias is omitted for simplicity. Often, a nonlinear (activation) layer is added after the convolution layer, usually rectified linear unit (ReLU). The activation layer applies the function *f(x)=max(0,x)* to all the values from the convolution layer. This process increases the nonlinear properties of the model and the overall network without affecting the receptive fields of the convolution layer. In this way, it resolves the vanishing problem compared with training traditional multilayer NNs with backpropagation. Pooling layer combines the output of neuron clusters in the convolution layer into a single neuron [56]. This is sometimes achieved by using max, sum, or average pooling, which consider the maximum, sum, or average value from each cluster of neurons, respectively [61]. FC layers connect the neurons in the previous layer to the neuron in the final layer by translating input image into a single vector for classification. This layer holds the filter that is used to determine the class of the input image. The output with the highest value is assigned the class label. The main benefit of a CNN is that during backpropagation, the network has to adjust a number of parameters in the filter with techniques such as gradient decent, which drastically reduce the connections of the CNN architecture.

**Figure 9:**
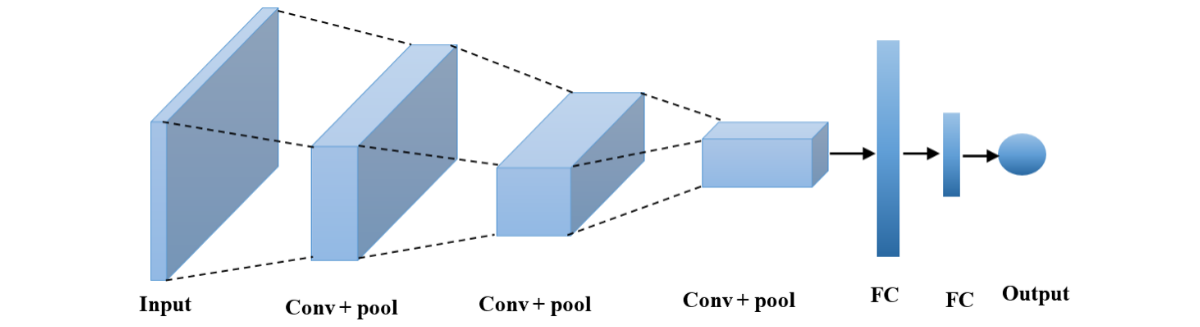
Structure of convolutional neural network with 3 convolution and pooling (conv+pool) layers and 2 fully connected (FC) layers.

In Figure 9, the general architecture of a simple CNN is presented, which shows the input, convolution+pooling, FC layer, and output layer. The convolution+pooling is responsible for feature extraction. The FC layer acts as a classifier on top of the features and assigns a probability score for the input image to define the output. The input to the convolution layer is an *m* *× m × r* image, where *m* is the height and width of the image and *r* is the number of channels. *k* is the filters (or kernels) in each convolution layer of size *n* *× n × q*, where *n* is smaller than the dimension of the image and *q* can be the same as *r*. The dimension of *k* can be *m - n + 1* which form the size of the filter (a locally connected structure). Each map is then subsampled with mean or max (*f(x)=max(0,x)*) pooling over contiguous region (*x*); additive bias and sigmoidal nonlinearity is applied to each feature map.

FC layer represents the feature vector of the input, a composite and aggregated information from all the convolution+pooling layers. Each node in the FC layer learns its own set of weights on all of the nodes in the layer below it. The final feature vector is used to predict the input image.

### Deep Boltzmann Machine

**Figure 10:**
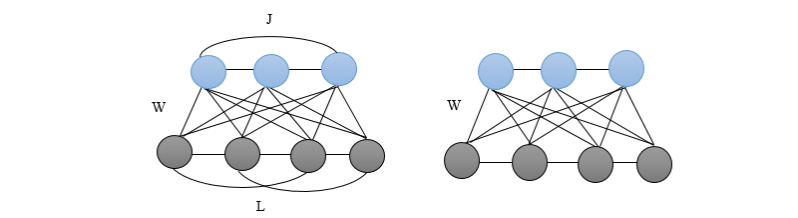
Left: A general Boltzmann machine. The top layer represents a vector of stochastic binary “hidden” features and the bottom layer represents a vector of stochastic binary “visible” variables. Right: A restricted Boltzmann machine with no hidden-to-hidden and no visible-to-visible connections. Where L, J, and W represent the visible layer, hidden layer, and connection weight between the layers respectively.

A Boltzmann machine (BM) is a network of symmetrically coupled stochastic visible and hidden units. The first diagram in Figure 10 shows the structure of BM, where the labels W, L, and J represent visible-to-hidden, visible-to-visible, and hidden-to-hidden symmetric interactions, respectively. BM model is suitable for modeling and extracting latent semantic representations from a large unstructured collection of documents [62]. The original algorithm for BM requires randomly initialized Markov chains to achieve equilibrium distributions to evaluate the data-dependent and data-independent expectations in a connected pair of binary variables [63]. Learning procedure is very slow in practice using this system [62]. To achieve an efficient learning, restricted Boltzmann machine (RBM) was created, which has no connections between hidden units [64]. The second diagram in Figure 10 shows a simple architecture of RBM with connections between neurons. A beneficial feature of RBM is that the conditional distribution over the hidden units factorizes, given the visible units. This makes inferences tractable as the RBM feature representation is taken to be a set of marginal posterior distributions obtained by directly maximizing the likelihood. Furthermore, 2 main DL frameworks in this category that have been presented in literatures are DBM and DBN [42].

The architecture of DBM NN is similar to RBM but with more hidden variables and layers. DBM architecture has entirely undirected connections between neurons within all layers [62]. The right image in Figure 10 shows the architecture of a simple DBM NN for 1 visible layer and 1 hidden layer. It has undirected connections between all layers of the network, but not within the neurons in a layer. For training a DBM, a stochastic maximum probability–based algorithm is usually applied to maximize the lower bound of the probability. This is because calculating the distribution over the posterior hidden neurons, given the visible neurons, cannot be achieved by directly maximizing the likelihood because of the interactions between the hidden neurons.

Implementation of DBM is remarkable as DBM has the capability to learn internal representations that become increasingly complex, which is regarded as a promising way of solving recognition problems. Moreover, in cases of semisupervised learning, high-level representations can be built from very limited labeled data and large supply of unlabeled inputs can then be used to fine-tune the model for specific task. In addition, to enable DBM propagate uncertainty and hence deal more robustly with ambiguous inputs, it can incorporate top-down feedback, in addition to an initial bottom-up pass.

### Deep Belief Network

**Figure 11:**
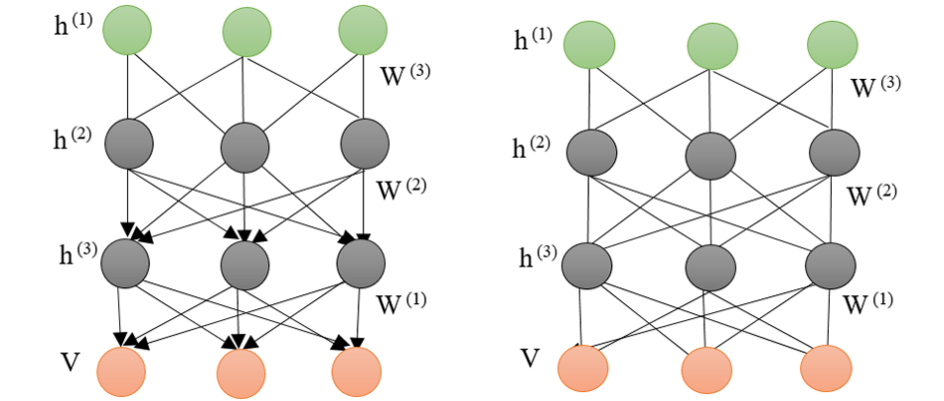
Left: A 3-layer deep belief network. Right: A stack of modified restricted Boltzmann machine constructed to create a deep Boltzmann machine. V: visible vector; h: a set of hidden neurons; w: connections.

DBN is another variant of RBM, where the multiple hidden layers can learn by treating the hidden output of one RBM as the input data for training the next layer of RBM [63,64]. It has undirected connections between its top 2 layers and directed connections between all its subsequent layers. The training strategy is greedy layer wise, which is performed when training the DBN using unsupervised learning and adjusting its parameters based on the expected output. The left diagram in Figure 11 illustrates the architecture of DBN with 3-layer configuration showing visible-to-hidden and hidden-to-hidden symmetric connections. The structure comprises several hidden layers of neurons, which are trained using backpropagation algorithm [65,66].

From Figure 11, the connection units in the DBN architecture is between each neuron in a layer with each neuron in the next layer; however, unlike RBM, there are no intraconnections among neurons within each layer.

### Trends in Deep Learning Methods

**Figure 12:**
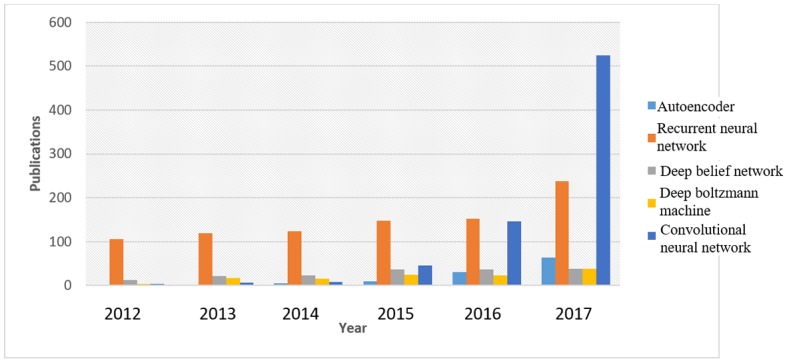
Research publications in different category of deep learning methods. These statistics are obtained from PubMed database by searching for publications containing any of the deep learning method in title or abstract.

**Figure 13:**
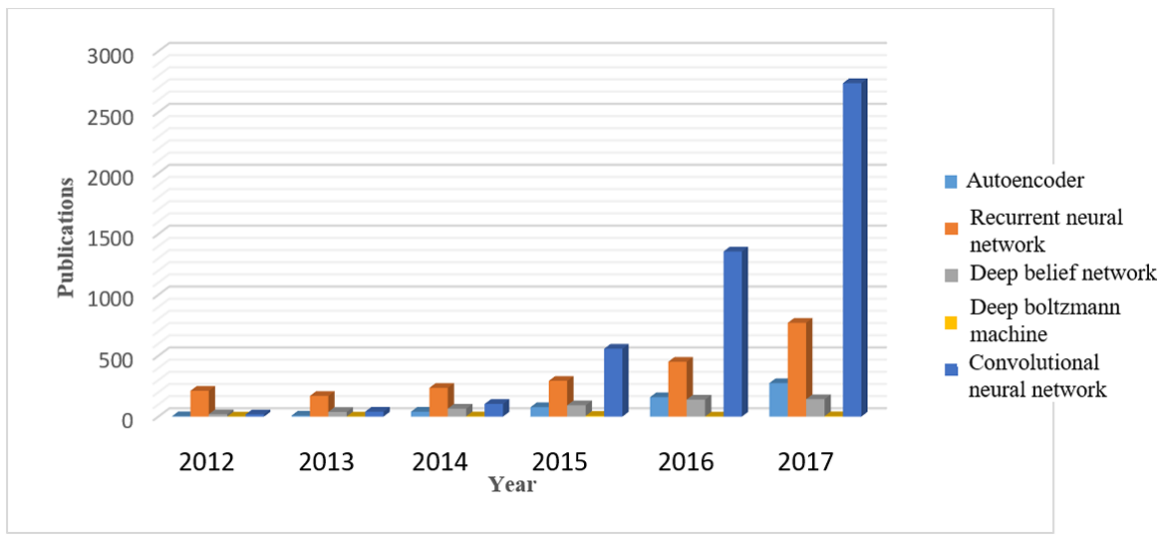
Publications distributions for 5 years in different categories of deep learning methods. These figures are extracted from the Institute of Electrical and Electronics Engineers database of papers from conferences and journals and magazines by using advanced query to search in publication title and abstract containing any of the deep learning methods ((“Publication Title”: Autoencoder) OR (“Abstract”: Autoencoder)).

The use of different DL techniques is proliferating into more domains in biomedical engineering applications. This is because of the achievements recorded in previously implemented applications. Figures 12 and 13 describe the trends in the use of different methods of DL over 5 years, from 2012 to 2017. The purpose of constructing the trend is to observe the implementation of DL methods over a period of time, and the choice of publication was based on implemented DL methods without any specific application domain. These statistics are obtained from 2 different sources, PubMed database and the Institute of Electrical and Electronics Engineers archive. Both Figures 12 and 13 show similarity in the pattern of increasing growth in the use of DL.

The observable pattern in both Figures 12 and 13 shows that RNN and CNN have a steady increase in application over the years, with CNN exhibiting tremendous growth rate. This can be attributed to the success recorded in image data and the many available variants of the model. Positron emission tomography and CT scan image processing are at the forefront of many health care applications. CNN has provided the needed processing techniques required to achieve expected performance. The growth rate in the application of this technique is expected to continue as more biomedical image applications will switch to this technique. Nevertheless, the growth rate is expected to slow down after a while as many applications would have migrated to this technique. Another DL method that has also shown promising performance is AE. The steady increase in the number of publications in Figure 12 and 13 indicates the successful implementation results and efficiency. DBN and BM have the least progression. Despite the small positive difference between successive years, the major challenge is in training the NN, which is computationally expensive. Another reason for the low rate of application is because of its combination with other methods and as it is sometimes implemented as a preprocessing phase. The graph in Figure 12 is obtained by searching for the DL techniques in publication title and abstract from PubMed. The result returns graphical statistics of publications grouped by years. An advanced search technique is applied to obtain Figure 13, where the query method is similar to the previous approach, but the query string is applied in title and abstract search fields. Therefore, the final query becomes ((*Publication Title*: Autoencoder) OR (*Abstract*: Autoencoder)). However, to get the total publication for each year, a manual filtering is used to get the distribution. The total publication is made up of both journals and conference articles.

### Comparative Analysis of Deep Learning Methods

The results reported in Figures 12 and 13 for different architectures of DL are based on the conceptual advantage of each method. Despite the fact that each DL model is more suitable for a particular kind of data or situation, there is however some relationship and cross-application of these methods. The fact that RNN and CNN are 2 commonly used DL techniques can be attributed to the need to solve data problems in the form and shape that only these techniques can handle effectively. In addition, most of the common data are either visual or time-dependent. Although CNN is a feedforward NN where information only flows in one (forward) direction, in RNN, the information flows back and forth as it operates on the principle of saving the output of the previous layer and feeding this back to the input to predict the output of the current layer. The principle of operation of CNN is influenced by the consecutive layer organization of the animal visual cortex. Therefore, it is designed to learn to recognize patterns across space. This makes CNN ideal for images (eg, 2D or 3-dimensional [3D] magnetic resonance images [MRI]), videos (eg, gait pattern, moving pattern in organs), and graphics (eg, tumor representation) to recognize features such as lines, edges, curves, and so on. On the contrary, RNN technique is suited to recognize patterns across time such that the information available now will subsequently influence what information will become available later. This makes it suitable for time series analysis such as sound (eg, heartbeat, speech), text (eg, medical records, gene sequence), and signals (eg, physiological signals such as electrocardiogram (ECG)).

There is a close comparison between DBN and DBM architectures. Although there is a diagrammatic similarity between DBN and DBM, similar to conventional DNN, both methods are deep, which means it is possible to create many hidden layers connecting the input and output unit. In addition, there is the presence of RBM in both architectures. However, they are qualitatively different. The connections between the layers in a DBN are directed, whereas it is undirected in DBM. The first 2 layers in a DBN is undirected connection of RBM; the subsequent layers are directed generative connections. However, in DBM, all the connections between the layers are undirected RBM. Another difference between these 2 techniques can be described in a general picture with the connected layers, where the connected layers in DBN function as sigmoid belief network but in DBM they are Markov random fields. DBN and DBM can be used to extract features from unprocessed physiological signals and image data (MRI) to reduce the size of features required for classification modeling. These models can also be applied as a generative model for human motions completion for fall detection or gait analysis.

The main function of AE architecture is to reconstruct the input data given to it. Therefore, if a vector *M* is given to AE, it tries to create *M*^i^*=h(g(M))* so that during training it obtains the parameter for *h* and *g* such that *M*^i^ is the same as *M*. In DBN and DBM, *h* and *g* exist between the input and hidden layers, although, to compute these functions a probabilistic (or Markov chains) approach is applied. However, unlike AE, there exists a special connection between *h* and *g* to make it a valid probabilistic model. In addition, although the model from AE ensures that the input and output are the same, DBN and DBM give a range of outputs for a given input it has been trained on because of its probabilistic principle. The similarity between AE and RBM model is to encode the visible layer with hidden layer in a constructive functional way; encoding the hidden layer with another hidden layer leads to stack AE and RBM (DBN and DBM). Considering the growing size of medical data, an efficient data coding with AE makes it possible to minimize memory requirements and reduce processing costs. Stacked AE can be applied in unsupervised feature learning for detection of tumors, cancers, or inflamed organs.

## Review of Deep Learning Implementation in Health Care

**Figure 14:**
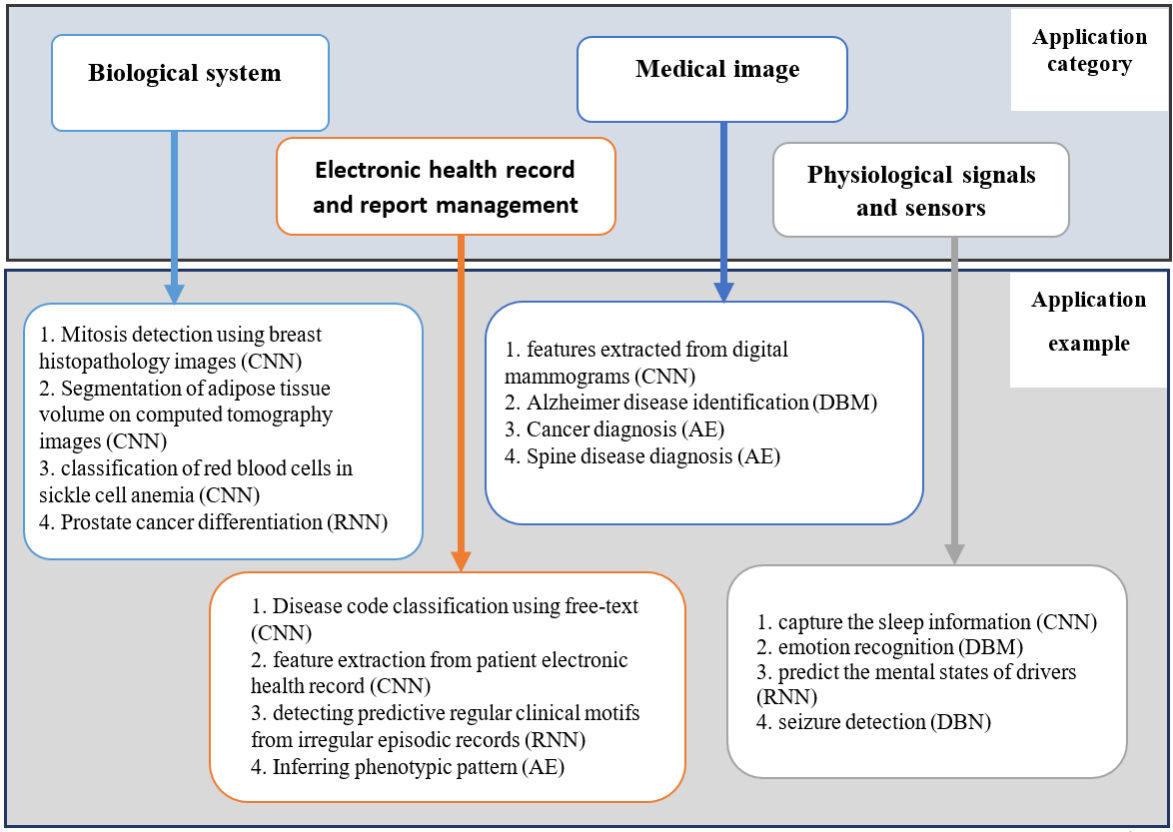
Descriptive summary of biomedical and health applications category and example implemented with deep learning methods: convolutional neural network (CNN), recurrent neural network (RNN), autoencoder (AE), deep boltzmann machine (DBM) and deep belief network (DBN).

This section reviews some health and biomedical areas that have successfully implemented DL techniques to create a model to solve specific task. We considered the DL methods discussed in the previous section and have presented a tabular representation of references for application in 4 areas: biological system, EHR and report management, medical image, and physiological signals and sensors. The tables provided represent the summary of applications of DL methods in each of these categories. The choice of literatures is selected from papers published between 2012 and 2018 that are related to health and medical applications. The purpose is to reveal some of the applications of DL methods that have been designed to solve biomedical-related tasks which initially have poor results with other techniques, such as handcrafted, or which seem unsolvable because of the complication of the task. Figure 14 shows an illustrative summary of application areas and implemented DL methods. It is an overview of the information presented in the tables provided. The figure is divided into 2 distinct blocks, application category and application example. The connection between the 2 blocks is created from the relationship between the content in the application category and application example.

### Biological System

Biological records such as DNA, RNA, genomes, sickle cell behavior, bacterial and viral multiplication, and mutation have features that make it possible to create a predictive model with DL algorithms to achieve performance exceeding human experts. Prediction could be in the form of discriminative gene identification, structured data in protein-protein interactions, image data from biological cell activities, drug composition–reaction profiling, and binding between DNA and proteins or between protein sequences. The structure of the data and expected goals determine the DL technique to be implemented. RNN, CNN, DBN, and AE methods have largely been applied in many aspects. Table 1 shows DL implementation in biological systems.

Biomedical analysis has benefited from CNN architecture. An example can be found in cell mitosis described in [67]. The CNN architecture primarily comprises 5 convolution layers, 4 max-pooling layers, 4 ReLUs, and 2 FC layers. The activation function used after each layer is ReLU and to avoid overfitting of the model, dropout layer was included after the first FC layer. In [68], a proposed CNN architecture for cell membrane and nuclei classification for breast cancer was developed, which comprised convolution and deconvolution sections. The framework mainly comprised multiple convolution layers, max-pooling layers, spatial pyramid pooling layers, deconvolution layers, upsampling layers, and trapezoidal LSTM. To achieve automatic red blood cell classification, [69] constructed a CNN architecture with alternating convolution and pooling operations to deal with nonlinear and complex patterns. A combination of 2 CNN techniques was constructed in [70], selection-CNN and segmentation-CNN, to achieve segmentation of adipose tissue volume on CT images.

Moreover, RNN architecture has been used in biomedical process to efficiently model problems with sequence and time feature. Sequence-specific bias correction problem for RNA sequence data was addressed using RNN architecture to model nucleotide sequence without predetermined sequence structures [75]. A variant of combined CNN and LSTM was created for prostate cancer identification with Gleason score of 7 [76]. The model assesses the correlation between histopathology images and genomic data with disease recurrence in prostate tumors to identify prognostic biomarkers within tissue by modeling the spatial relationship from automatically created patches as a sequence within the tissue. In [77], biomedical LSTM and conditional random fields (CRF) were combined to create a relationship between biological entities for trigger detection. The method is based on the sequence annotation that does not require initial complex feature engineering but only requires a simple labeling mechanism to complete the training.

The synergistic effect of drug combination is one of the most desirable properties for treating cancer. A method based on DBN was created to predict drug synergy from gene expression and pathway and ontology fingerprints [78]. A multiclassifier DBN technique was proposed to detect mitotic cells in hematoxylin and eosin-stained images using step by step refinement of segmentation and classification stages [79]. The multiclassifier DBN algorithm segments cell nuclei from background stroma. Critical proteins exhibiting dramatic structural changes in dynamic protein-protein interactions networks were identified in [80] using a DBN framework; the reconstruction errors and the variabilities across time were analyzed in the biological process. In [81], a DBM framework called stacked RBM was proposed to analyze the RNA-seq data of Huntington disease. In addition, the framework was able to screen the key genes during the Huntington disease development. The initial step for the framework was to select disease-associated factors with different time period datasets according to the differentially activated neurons in hidden layers. Then, the disease-associated genes were selected according to the changes of the gene energy at different time periods.

Furthermore, AE technique has been considered as a method to delineate signals from noise in imaging to enhance image quality. In [83], a proposed deep count AE to denoise small cytoplasmic RNA sequence (scRNA-seq) datasets was created. The model takes the count distribution, dispersion, and sparsity of the data into account using a negative binomial noise model with or without zero inflation. The technique was capable of capturing nonlinear gene-gene dependencies. Another design of AE model proposed was called stacked denoising AE for multilabel learning [84]. The purpose was to facilitate gene multifunction discovery and pathway completion. The technique can capture intermediate representations robust to partial corruption of the input pattern for cancer research, pathway analysis, and gaining insight into the underlying biology associated with cancer. A gene superset AE [85], a multilayer model with the incorporation of a priori defined gene sets, retains the crucial biological features in the latent layer. The method introduced the concept of gene superset, an unbiased combination of gene sets with weights trained by AE, where each node in the latent layer is a superset. Furthermore, to analyze the transcriptomic heterogeneities at the single cell level, a deep variational AE technique for scRNA-seq data was proposed [86]. The technique is a deep multilayer generative model for unsupervised dimension reduction and visualization of scRNA-seq data. The AE technique can explicitly model the dropout events and find the nonlinear hierarchical feature representations of the original data.

**Table 1:** Deep learning implementation in biological systems.

Reference	Task	Method	Remark
Saha et al, 2018 [67]	Mitosis detection	CNN^a^	The prediction model has an improved 92% precision, 88% recall, and 90% F-score over conventional machine learning methods
Saha et al, 2018 [68]	Cell membranes and nuclei classification	CNN	The identification model achieved predictive value of 98.33% accuracy and 6.84% false-positive rate which was comparable with human expert
Xu et al, 2017 [69]	Red blood cells classification	CNN	Framework classified sickle-shaped red blood cells in an automated manner with above 90% accuracy
Wang et al, 2017 [70]	Segmentation of adipose tissue	CNN	The model was tested on 2 datasets; the accuracy produced 95.8% and 96.8% for computed tomography slice selection-CNN and fat pixel segmentation-CNN, respectively
Xu et al, 2017 [71]	Classification, segmentation of tissue	CNN	Outcome generates patterns that reveal biological insights that have been verified by pathologist
Hughes et al, 2016 [72]	Reactivity to biological macromolecules	CNN	The model captured molecules that would have been missed by standard reactivity screening experiments
Song et al, 2018 [73]	Segmentation of cervical cytoplasm	CNN	Experimental results achieved an accuracy of 94.50% for nucleus region detection and a precision of 0.9143(SD 0.0202) and a recall of 0.8726(SD 0.0008) for nucleus cell segmentation
Gurcan et al, 2001 [74]	Detection of microcalcifications	CNN	The results demonstrated optimization of cost surface whose characteristics are not known
Han et al, 2015 [30]	Membrane bioreactor permeability	RNN^b^	Simulation and experimental results demonstrate the reliability and effectiveness of the proposed intelligent detection system
Zhang et al, 2017 [75]	Sequence-specific correction for RNA	RNN	RNN-based bias correction method compares well with the state-of-the-art sequence-specific bias correction method
Ren et al, 2018 [76]	Prostate cancer differentiation	RNN	Their study demonstrates that prostate cancer patients with Gleason score of 4+3 have a higher risk of disease progression and recurrence compared with prostate cancer patients with Gleason score of 3+4
Wang et al, 2018 [77]	Detecting biomedical event trigger for protein and gene	RNN	F-score potentially reached about 80%, which is better than comparative experimental methods
Chen et al, 2018 [78]	Effective drug combination	DBN^c^	Predict effective drug combination from gene expression and pathway and ontology fingerprints properties for treating cancer
Beevi et al, 2017 [79]	Cell mitosis detection	DBN	The algorithm provides improved performance compared with other state-of-the-art techniques with average F-score of 84.29% for the MITOS^d^ dataset and 75% for the clinical dataset from Regional Cancer Centre
Zhang et al, 2014 [80]	Identification of critical proteins	DBN	The results of comparison showed that DBN had higher reconstruction rate compared with baseline methods and more proteins of critical value to yeast cell cycle process were identified
Jiang et al, 2016 [81]	Huntington disease identification	DBM^e^	Results demonstrate that the model can detect important information for differential analysis of time series gene expression datasets
Ghasemi et al, 2017[82]	Biological activity prediction	DBN	The output of the model demonstrated significant superiority to traditional neural network with random parameters
Eraslan et al, 2019 [83]	Single cell RNA-seq denoising	AE^f^	Outperforms existing methods for enhancing biological discovery and data imputation in terms of quality and speed
Guan et al, 2018 [84]	Gene function annotation	AE	The model can capture intermediate representations to partial corruption of input pattern and generate low-dimensional codes superior to conditional dimension reduction tools
Chen et al, 2018 [85]	Genomics functional characterization	AE	Retains sufficient biological information with regard to tumor subtypes and clinical prognostic significance. Provides high reproducibility on survival analysis and accurate prediction for cancer subtypes
Wang et al, 2018 [86]	Visualization of single cell RNA- seq	AE	Reconstructs the cell dynamics in preimplantation embryos and identifies several candidate marker genes associated with early embryo development
Maggio et al, 2018 [87]	Prognostic profiling for survival prediction	AE	The embedding technique can be used to better stratify patients’ survival
Hu et al, 2018 [88]	Prediction of drug-likeness	AE	The classification accuracy of drug-like/nondrug–like models are 91.04% on WDI-ACD^g^ databases and 91.20% on MDDR-ZINC^h^ database

^a^CNN: convolutional neural network.

^b^RNN: recurrent neural network.

^c^DBN: deep belief network.

^d^MITOS: mitosis detection in breast cancer histological images.

^e^DBM: deep Boltzmann machine.

^f^AE: autoencoder.

^g^WDI-ACD: world drug index-available chemicals directory.

^h^MDDR-ZINC: MDL drug data report-zinc compound.

### Health Record and Report Management

EHR stores patient’s data for improving health care and providing personalized treatment and historical account. This information can be in the form of radiological images, diagnosis, temporal event extraction, doctor health review, disease classification, demographic details, prescription, laboratory tests, and results. Over the years, these records have increased proportionally making it difficult and challenging for health workers and medical professionals to handle. Until the last decade, most approaches were based on statistical techniques and few attempts to use ML. Currently, those methods have become infeasible because of the large and increasing amount of these data. DL becomes imperative to be able to make meaning from these data. Table 2 presents some literature of DL implementation in EHR.

CNN techniques have been adapted to solve some task related to EHR to achieve better health management system. Feature engineering remains a major bottleneck when creating predictive systems for EHRs [89]. Word embedding from discharge notes combined with CNN was applied to disease code classification [90]. The model was based on a 1-layer CNN with a filter region size of 1 to 5 to increase comparability with traditional ML techniques. One study [91] proposed a CNN method for phenotyping from patients’ EHRs. For the initial setup, every patient’s record was represented as a temporal matrix with time on one dimension and event on the other dimension. A 4-layer CNN model was created for extracting phenotypes and performing prediction. The first layer comprised the temporal matrix. The second layer was a one-side convolution layer that could extract phenotypes from the first layer. The third layer was a max-pooling layer introducing sparsity on the detected phenotypes so that only those significant phenotypes would remain. The fourth layer was an FC softmax prediction layer.

Similarly, RNN methods have also been applied to solve problems relating to EHR to understand symptoms and achieve improved health care quality and personalized medication. A combination of bidirectional LSTM and CRF network has been implemented to recognize entities and extract relationship between entities in EHR [93]. To improve the model, multitask learning was included to handle hard parameter sharing, parameter regularization, and task relation learning. In addition, another LSTM and CRF to recognize clinical entities from Chinese EHR data was proposed [94]. Character embedding and segmentation information were used as features to be able to semantically understand diagnoses, tests, body parts, symptoms, and treatments. In [95], LSTM method was used for structured prediction in clinical text and in [96], RNN frameworks were explored and proved to be significantly better than CRF models. In [97], the LSTM implementation was for a single data structure that could be used for many predictions, rather than requiring custom, hand-created datasets for every new prediction. This approach represented the entire EHR in temporal order, which represents the event in a patient’s timeline.

In addition, RBM and AE DL techniques have been constructed to handle challenging tasks found in EHRs to provide solutions or serve as a preprocessing step for another technique. A DBN framework was applied to predict the risk of osteoporosis from heterogeneous EHR for monitoring bone disease progression [104]. The framework is capable of pinpointing the underlying causes of the disease to assess the risk of a patient in developing a target disease and discriminating between patients suffering from the disease for the purpose of selecting risk factor of the disease. In [105], 2 novel modifications to DBN training was proposed to address the challenges and exploit properties that are peculiar, if not exclusive, to medical data. First, a general framework was examined for prior knowledge to regularize parameters in the topmost layers. Second, a scalable procedure was described for training a collection of NNs of different sizes but with partially shared architectures. AE model was developed for handling computational task and analysis of EHR [107-109]. In [109], the challenge of traditional supervised learning approach for inferring precise phenotypic patterns was addressed. Conventionally, an expert designates which pattern to look for (by specifying the learning task and the class labels) and where to look for them (by specifying the input variables). Although this is appropriate for individual tasks, this approach scales poorly and misses the patterns. Unsupervised feature learning with AE was able to handle these limitations by identifying patterns (or *features*) that collectively form a compact and expressive representation of the source data, with no need for expert input or labeled examples.

**Table 2:** Deep learning implementation in electronic health records and medical report management.

Reference	Task	Method	Remark
Wickramasinghe et al, 2017 [89]	Extract features from medical records	CNN^a^	It achieves superior accuracy compared with traditional techniques to detect meaningful clinical motifs and uncovers the underlying structure of the disease
Lin et al, 2017 [90]	Disease code classification	CNN	The method had a higher testing accuracy (mean AUC^b^=0.9696; mean F-score=0.9086) than traditional NLP^c^-based approaches (mean AUC range 0.8183-0.9571; mean F-score range 0.5050-0.8739)
Cheng et al, 2016 [91]	Risk prediction of chronic congestive heart failure	CNN	The model performance increases the prediction accuracy by 1.5% when 60% training data were used and 5.2% when it is 90% training data
Zeng et al, 2017 [92]	MobileDeepPill: Recognition of unconstrained pill image	CNN	DL^d^-based pill image recognition algorithm won the first price of the NIH^e^ NLM^f^ Pill Image Recognition Challenge
Li et al, 2018 [93]	Extraction of adverse drug events	RNN^g^	The DL model achieved a result of F-score=65.9%, which is higher than F-score=61.7% from the best system in the MADE^h^1.0 challenge
Zhang et al, 2018 [94]	Identify clinical named entity	RNN	CRF^i^ and bidirectional LSTM^j^-CRF achieved a precision of 0.9203 and 0.9112, recall of 0.8709 and 0.8974, and F-score score of 0.8949 and 0.9043, respectively
Jagannatha et al, 2016 [95]	Prediction based on sequence labeling	RNN	Prediction model improved detection of the exact phrase for various medical entities
Jagannatha et al, 2016 [96]	Extraction of medical events	RNN	Cross-validated microaverage of precision, recall, and F-score for all medical tags for gated recurrent unit–documents are 0.812, 0.7938, and 0.8031, respectively, which are higher than other methods
Rajkomar et al, 2018 [97]	Representation of patients’ record	RNN	Achieved high accuracy for tasks such as predicting in-hospital mortality, prolonged length of stay, and all of a patient’s final discharge diagnoses
Hou et al, 2018 [98]	Extraction of drug-drug interaction	RNN	DL can efficiently aid in information extraction (drug-drug interaction) from text. The F-score ranged from 49% to 81%
Choi et al, 2015 [99]	Predicting clinical events	RNN	On the basis of separate blind test set evaluation, the model can perform differential diagnosis with up to 79% recall, which is significantly higher than several baselines
Choi et al, 2016 [100]	Detection of heart failure onset	RNN	When using an 18-month observation window, the AUC for the RNN model increased to 0.883 and was significantly higher than the 0.834 AUC for the best of the baseline methods
Volkova et al, 2017 [101]	Forecasting influenza-like illness	RNN	LSTM model outperformed previously used models in all metrics, for example, Pearson correlation (0.79), RMSE^k^ (0.01), RMSPE^l^ (29.52), and MAPE^m^ (69.54)
Yadav et al, 2016 [102]	Patient data deidentification	RNN	The proposed approach achieved best performance, with 89.63, 90.73, 90.18 for recall, precision, and F-score, respectively
Hassanien et al, 2013[103]	Classification of diagnoses	RNN	Models outperformed several strong baselines, including a multilayer perceptron trained on hand-engineered features
Li et al, 2014 [104]	Identifying informative risk factors and predicting bone disease	DBN^n^	Proposed framework predicted the progression of osteoporosis from risk factors and provided information to improve the understanding of the disease
Che et al, 2015 [105]	Detection of characteristic patterns of physiology	DBN	The empirical efficacy of the technique was demonstrated on 2 real-world hospital datasets and the model was able to learn interpretable and clinically relevant features
Tran et al, 2015 [106]	Harness electronic health record with minimal human supervision	DBM^o^	The model achieved F-scores of 0.21 for moderate-risk and 0.36 for high-risk, which are significantly higher than those obtained by clinicians and competitive with the results obtained by support vector machine
Miotto et al, 2016 [107]	Predict future of patients	AE^p^	Results significantly outperformed those achieved using representations based on raw electronic health record data and alternative feature learning strategies
Lv et al, 2016 [108]	Clinical relation extraction	AE	The proposed model is validated on the dataset of i2b2 2010. The DL method for feature optimization showed great potential
Lasko et al, 2013 [109]	Inferring phenotypic patterns	AE	The model distinguished the uric acid signatures of gout and acute leukemia despite not being optimized for the task

^a^CNN: convolutional neural network.

^b^AUC: area under the curve.

^c^NLP: natural language processing.

^d^DL: deep learning.

^e^NIH: national institutes of health.

^f^NLM: national library of medicine.

^g^RNN: recurrent neural network.

^h^MADE: medication and adverse drug events.

^i^CRF: conditional random fields.

^j^LSTM: long short-term memory.

^k^RMSE: root mean square error.

^l^RMSPE: root mean square percentage error.

^m^MAPE: mean absolute percentage error.

^n^DBN: deep belief network.

^o^DBM: deep Boltzmann machine.

^p^AE: autoencoder.

### Medical Image

The success of DL algorithms for image segmentation, localization, classification, and recognition task in recent years is timely with remarkable increase in medical image data. Analysis of these data has become an active field, partly as image data are easier for clinicians to interpret and they are relatively structured and labeled. There have been reported accuracies in some publications for detecting a range of anomalies such as malignant tumor, breast mass localization, recognition of pathology (organ parts), infectious diseases, and coronary artery stenosis classification. CNN and AE have been commonly implemented to solve challenging medical image problems. This is because the structure of these DL methods makes it possible to learn salient features from the data to create different levels of abstraction to achieve the required result. Table 3 lists some areas in medical image that have implemented DL solution. The performance exceeds ML techniques such as SVM and random forest classifiers.

The use of statistical pooling strategy was crafted into building CNN model in [110], with feature extraction at different convolutional layers and a multivariate classifier trained to predict which tumor contained occult invasive disease. Another pooling technique is stochastic pooling for alcoholism detection [111]. In [112], a CNN method that uses multiple patch sizes and multiple convolution kernel sizes was proposed to acquire multiscale information about each voxel to achieve accurate segmentation of tissue in brain images. Chest diseases are very serious health problems in the life of people. One study [113] presented CNN model for diagnosis of chest diseases. The designed model was trained and tested using chest x-ray images containing different diseases.

Moreover, [115] presented a modified DBN technique to minimize the computation load from 3D ultrasound data for the first trimester of pregnancy, which is an important parameter in prenatal screening. The technique converts the sagittal plane into a symmetry plane and axis searching problem. Feature extraction requires technical and task-specific approach such as neuroimaging, which contains features for diagnosing diseases. A DBM technique was proposed for a high-level hierarchical latent and shared feature representation from 3D patch neuroimaging modalities [116]. An integrated visual and textual multimodal image retrieval approach was created for cancer clinical practice and research with DBM method [117]. In addition, the DBM method can be used to extract volumetric representations from 3D brain image for classification of sensorimotor activities. The weights in the higher level of the architecture show spatial patterns that can identify specific tasks and the third layer represents distinct patterns or codes. In [118], a deep generative shape model–driven level set method was developed and evaluated to address automatic heart motion tracking to minimize radiation-induced cardiotoxicity. The proposed heart motion tracking method made use of MRI image sequences that characterize the statistical variations in heart shapes. This heart shape model was established by training a 3-layered DBM to characterize both local and global heart shape variations.

**Table 3:** Application of deep learning techniques in medical images.

Reference	Task	Method	Remark
Shi et al, 2018 [110]	Occult invasive disease prediction	CNN^a^	The performance result exceeded handcrafted computer vision technique that was designed with prior domain knowledge. It achieved operating characteristic curve of 0.70 and 95% CI
Wang et al, 2018 [111]	Alcohol detection	CNN	The method used multiple images in the experiment and achieved 96.88% sensitivity, specificity of 97.18%, and accuracy of 97.04%
Moeskops et al, 2016 [112]	Tissue segmentation	CNN	The result demonstrates accurate segmentation in all datasets and the robustness to different age and acquisition protocol
Abiyev et al, 2017 [113]	Chest disease detection	CNN	Demonstrate accurate classification of chest pathologies such as chronic obstructive pulmonary disease, pneumonia, asthma, tuberculosis, and lung diseases in chest x-rays
Liu et al, 2016 [114]	Food image recognition for dietary assessment	CNN	These results outperformed all other reported work such as DeepFoodCam using UEC-256 and Food-101 dataset
Nie et al, 2017 [115]	Detection of standard sagittal plane in pregnancy	DBN^b^	The model provides knowledge to avoid unnecessary massive searching and corresponding huge computation load
Zhang et al, 2016 [116]	Benign and malignant breast tumors differentiation	DBM^c^	Results showed that the deep learning method achieved better classification performance with an accuracy of 93.4%, a sensitivity of 88.6%, a specificity of 97.1%, and an area under the receiver operating characteristic curve of 0.947
Cao et al, 2014 [117]	Cancer clinical practice and research	DBM	Experimental results with large volume of real-world medical images showed that multimodal approach is a promising solution for the next generation medical image indexing and retrieval system
Wu et al, 2018 [118]	Tracking motion of the heart	DBM	Heart shape model that characterizes the statistical variations in heart shapes present in a training dataset for tracking motion of the heart
Jang et al, 2017 [119]	Four sensorimotor classification	DBM	Identified task-specific (left hand clenching, right hand clenching, auditory attention, and visual stimulus) features and classification of functional/structural magnetic resonance imaging volumes
Suk et al, 2014 [120]	Alzheimer disease identification	DBM	Achieved maximum accuracy of 95.35%, outperforming other computing methods
Khatami et al, 2016 [121]	Extract high-level features from medical images	DBN	Experimental results show that the proposed model improves about 0.07% performance compared with other models
Zhang et al, 2019 [122]	Discovering hierarchical common brain networks	DBN	Three hierarchical layers with hundreds of common and consistent brain networks across individual brains were successfully constructed
Hu et al, 2019 [35]	Cancer diagnosis	AE^d^	Diagnosed malignant mesothelioma, a rare but aggressive cancer because of its composite epithelial/mesenchymal pattern
Uzunova et al, 2018 [123]	Pathology detection	AE	Experiments on 2-dimensional and 3-dimensional datasets show that the approach is suitable for detection of pathologies and deliver reasonable dice coefficient result
Lee et al, 2018 [124]	Benign and malignant tumor classification	AE	The results show that when deep learning algorithm is applied on sonograms after intensity inhomogeneity correction, there is a significant increase of the tumor classification accuracy
Seebock et al, 2018 [125]	Age-related macular degeneration classification	AE	Used markers to classify early and late age-related macular degeneration cases. The model yields an accuracy of 81.40%
Wang et al, 2019 [126]	Spine disease diagnosis	AE	Achieved higher localization accuracy, low model complexity, and without the need for any assumptions about visual field in computed tomography scans
Malek et al, 2017 [127]	Image description for visually impaired	AE	Fusing a set of AE-learned features gave higher classification rates with regard to using the features individually
Zhang et al, 2016 [128]	Histopathological images analysis	AE	The method effectively combined the strength of multiple features adaptively as inputs and achieves 91.67% classification accuracy
Xia et al, 2016 [129]	Human attention process	AE	Experimental results on several benchmark datasets show that in accordance with different inputs, the network can learn distinct basic features for saliency modeling in its encoding layer
Mano et al, 2018 [130]	Chronic back pain detection	AE	Experimental results from patients in the United Kingdom and Japan (41 patients, 56 controls), achieved accuracy of 63%, with 68% in cross-validation of all data

^a^CNN: convolutional neural network.

^b^DBN: deep belief network.

^c^DBM: deep Boltzmann machine.

^d^AE: autoencoder.

Furthermore, preprocessing with AE can handle noise in images for tumor detection and intensity inhomogeneity correction before performing classification. A combination of multiple techniques can also be constructed, for example, [119] considered point-wise gated BM and RBM to identify tumor from image data. In [123], it was demonstrated that conditional variational AE can learn the reconstruction and encoding distribution of different variabilities of 2D and 3D images to learn the appearance of pathological structures. Consequently, preprocessing of image can be done to highlight features that are fed into DL architecture to increase distinguishing tumor accuracy [124]. Most applications of DL methods for diagnosis and classification of diseases require that the images are marked for training; however, because of the limitation of marked entities and training examples, supervised training does not scale well. Furthermore, a multiscale deep denoising AE was constructed without the constraint of prior definition for the identification of anomalies occurring frequently in retinal optical coherence tomography image data [125]. Qualitative analysis of these markers shows predictive value in the task of detecting healthy, early, and late age-related muscular degeneration. A combination of contextual features from deep stacked sparse AE (SSAE) and structured regression forest for vertebrae localization and identification was created to overcome handcrafted and low-level features of spine structure [126]. The method employs SSAE to learn deep contextual features by building larger-range input samples to improve the contextual discrimination ability.

### Physiological Signals and Sensors

Advancement in sensing technology has made it possible to acquire and analyze signals from patients for monitoring mental state, heart condition, and disease diagnosis. An important process for achieving accurate and high-performance model depends on feature extraction and feature selection. DL algorithms have recorded successful achievement in modeling physiological signals and sensor data with better accuracy compared with traditional ML techniques. Table 4 presents some implementations of DL methods described in literatures using sensors and physiological signals for health care management. One significant characteristic of this kind of medical data is that they are sequential and time-dependent. Therefore, a technique for creating a model should not only take into consideration the shape of the data (spatial features), but also the time factor (temporal features). Skin conductance sensor, microphone for speech sequence, blood volume pulsation, electroencephalogram (EEG), photoplethysmogram (*PPG*), ECG, and so on are some examples of data in this category. RNN and its variants have been predominantly applied in this domain as it is able to model the data in the context of time and sequence to reveal hidden features. DBN and AE have also been implemented to solve problems with this kind of data.

Many variations of CNN techniques have been created to achieve enhanced performance and there are some optimization techniques that have been applied to achieve better accuracy. In [131], a technique based on CNN called fast discriminative complex-valued CNN for automatic sleep stage classification was developed. The method can capture the sleep information hidden inside EEG signals and automatically extract features from the signal. The constructed CNN method eliminated the need for deep digital signal processing skills, which usually serve as preprocessing phase for most signal operations. The time series plots generated directly from accelerometer and gyroscope signals were employed for classification of human activity and exercise detection [132]. The classification task was achieved with CNN, where the generated signals were formatted into image dimension. Estimation of brain activation with CNN was addressed in [133], where the temporal region was modified with unscented Kalman filter and a corresponding unscented smoother to observe inference relation of task-specific brain network. The CNN model parameters were estimated using expectation-maximization algorithm to exploit the partial linearity of the model. Moreover, the initial challenge encountered in building a model is data preprocessing and setup. Often data are irregular, inconsistent, and sometimes contain irrelevant information. Therefore, the preprocessing bottleneck for the creation of 2 mental state classification models for drivers from EEG signals was handled with CNN and deep residual learning [38]. The model contains 8 layers: the input layer, 3 convolutional layers, a pooling layer, a local response normalization layer, an FC layer, and the output layer.

**Table 4:** Deep learning technique for sensors and physiological signal task.

Reference	Task	Method	Remark
Zeng et al, 2018 [38]	Predict mental states of drivers	CNN^a^	Predicted the mental states of drivers from electroencephalography (EEG) signals using 2 mental state classification models called EEG-Conv and EEG-Conv-R
Zhang et al, 2017 [131]	Sleep stage classification	CNN	The total accuracy and kappa coefficient of the proposed method are 92% and 0.84, respectively
Veiga et al, 2017 [132]	Human activity classification	CNN	The exercises could be recognized with 95.89% accuracy
Lenz et al, 2011 [133]	Interactions in human brain	CNN	Result showed regions of human brain affected by interactions and activities
Murad et al, 2017 [32]	Human activity recognition	RNN^b^	Experimental results showed that the proposed method outperforms methods employing conventional machine learning algorithms, such as support vector machine and k-nearest neighbors
Liu et al, 2016 [134]	Predicting driving fatigue	RNN	Identified brain dynamics in predicting car driving fatigue. The model was evaluated using the generalized cross-subject approach
Yu et al, 2015 [135]	Human action classification	RNN	Real-time human action classification for recording and regenerating both action sequences and action classification tasks from continuous signal
Vakulenko et al, 2017[136]	Human body motion analysis	RNN	The method generated missing information from human body motions from sparse control marker settings
Mo L et al, 2016; Ordóñez et al, 2016 [137,138]	Human physical activity recognition	RNN	The model can recognize 12 types of activities and the accuracy rate was 81.8%. CNN was applied for feature extraction and long short-term memory model for the human physical activity recognition
Mathews et al, 2018 [36]	Cardiac arrhythmias diagnosis	DBM^c^	Single-lead electrocardiogram model detected cardiac abnormalities which was comparable with human expert
Chu et al, 2018 [40]	Recovery motor imagery	DBN^d^	Recognize and restructure the incomplete motor imagery in electroencephalogram signals for recovery motor imagery-based treatment
Turner et al, 2014 [139]	Seizure detection	DBN	High resolution biosensing multichannel model achieved personalized health monitoring
Chao et al, 2018 [140]	Emotion recognition	DBM	The results showed that the proposed framework outperforms other machine learning classifiers
Jindal, 2016 [141]	Heart rate monitoring	DBM	Technique was able to predict heart rate with a 10-fold cross-validation error margin of 4.88%
Hassan et al, 2018 [142]	Human activity recognition	DBN	The proposed approach outperformed traditional expression recognition approaches such as typical multiclass support vector machine and artificial neural network
Ruiz-Rodríguez et al, 2014 [141]	Blood pressure monitoring	DBM	Continuous noninvasive blood pressure monitoring system with performance higher than benchmark methods
Yuan et al, 2019 [144]	Epileptic seizures detection	AE^e^	Experimental results showed that the proposed model was able to achieve higher average accuracy and F-score of 94.37% and 85.34%, respectively
Jirayucharoensak et al, 2014 [145]	Detection of emotion	AE	The model provided better performance compared with support vector machine and Naive Bayes classifiers
Jokanović 2017 [146]	Human fall detection	AE	Experimental data were used to demonstrate the superiority of the model over principal component analysis method
Xia et al, 2018 [147]	Cardiac arrhythmia classification	AE	The results from the model (99.8% accuracy) showed that the classification performance of the proposed approach outperforms most of the state-of-the-art methods

^a^CNN: convolutional neural network.

^b^RNN: recurrent neural network.

^c^DBM: deep Boltzmann machine.

^d^DBN: deep belief network.

^e^AE: autoencoder.

Similarly, RNN methods have been effective in mining discriminative features from raw input sequences acquired from body-worn sensors. The use of deep RNN for building recognition models for human activities was proposed in [32]. It was capable of capturing long-range dependencies in variable-length input sequences. Moreover, the use of fuzzy logic with RNN to create a recurrent fuzzy NN increases adaptability and the bottleneck of regression problem to handle driving fatigue for preventing road accidents [134]. In addition, classification of multiple types of motion when observing a human action can be difficult because of the complex nature of the timescale signal. Therefore, [135] proposed supervised multiple timescale RNN architecture for handling the issue of action classification. To overcome the difficulty of setting the initial states, a group of slow context nodes, known as *classification nodes*, was created. The supervised model provides both prediction and classification outputs simultaneously. In [136], continuous-time RNN networks were considered as dynamic models for the simulation of human body motion. These networks comprise a few centers and many satellites connected to them. The centers evolve in time as periodical oscillators with different frequencies.

Furthermore, [40] developed a decoding scheme from a combination of Lomb-Scargle periodogram and DBN to recognize incomplete EEG signal data to solve the problem of motor imagery recovery for performing classification tasks such as heart rate variability. The use of a variety of representations and DBM algorithms was explored for seizure detection in high resolution, multichannel EEG data [138]. In addition, a DL framework based on improved DBN with glia chains (DBN-GCs) for handling emotion recognition task was constructed [140]. In the framework, DBN-GCs are employed for extracting intermediate representations of EEG raw features from multiple domains separately, as well as for mining interchannel correlation information by glia chains. The higher-level features describe time domain characteristics and frequency domain characteristics. The time-frequency characteristics are fused by a discriminative RBM to implement emotion recognition task. Owing to the increasing monitoring of heart rate through mobile phones and wearable devices, [141] presented a novel technique for accurately determining heart rate during intensive motion by classifying PPG signals obtained from mobile phones or wearable devices integrated with motion data obtained from accelerometer sensors.

Moreover, AE models have also been constructed to solve health and biomedical challenges using signals and sensors. For example, AE-based multiview learning was implemented to monitor and analyze multichannel EEG signals of epileptic patients to prevent complications caused by epileptic seizures [142]. The implemented approach was an end-to-end model that was able to jointly learn multiview features from both unsupervised multichannel EEG reconstruction and supervised seizure detection via spectrogram representation. In [145], the utilization of stacked hierarchical AE learning approach was proposed for automatic emotion recognition with nonstationary EEG signals. To alleviate overfitting problem, principal component analysis was applied to extract the most important components of the initial input. In addition, covariate shift adaptation of the principal components was implemented to minimize the nonstationary effect of EEG signal. In another similar implementation, a stacked AE was proposed to detect human fall [146]. The proposed approach automatically captures the intricate properties of signal from radar. To minimize false alarms in human fall detection, information from both the time-frequency and range domains was fused together.

## Challenges in Health Care for Deep Learning Applications

In spite of the all the impressive achievements and capabilities of DL discussed in the previous section, the technique is still in its infancy in biomedical and bioengineering applications. There are significant challenges that need to be resolved for DL to be able to handle the inherent medical and health care challenges. This section highlights some of the challenges confronting the implementation of DL methods.

### Medical Data Representation and Transformation

DL algorithms can make the most effective observations and predictions with the appropriate type and quantity of data. Currently, real-world medical data are in unstructured formats such as sequences (time series, audio and video signals, DNA, and so on), trees (XML documents, parse trees, RNA, etc), text data (symptoms description, tumor description, medical records), or combinations of any of these formats [148]. Unfortunately, the core of DL technique can only process numeric input data as eventually it is broken down to strings of zeros and ones for computing system. Some qualitative data are not easily converted into a usable format and processing can sometimes become complicated. Humans can easily process and make meaning of these data and when there is a simultaneous change, for example, in intensity and quantity, it can easily be understood and adjustment can be made with regard to the changes; an example is temperature and light. The representation of similar processes and conditions in DL requires a lot of encoding and thoughtful mathematical expressions in DR and transformation. Cross-domain feature learning algorithm based on stacked denoising AE has been considered for effective feature representation to describe data with multimodal property (eg, signals, image, video, and audio) [149]. A DL architecture that is capable of integrating multiple types of data concurrently is needed to handle some real-world situations.

**Figure 15:**
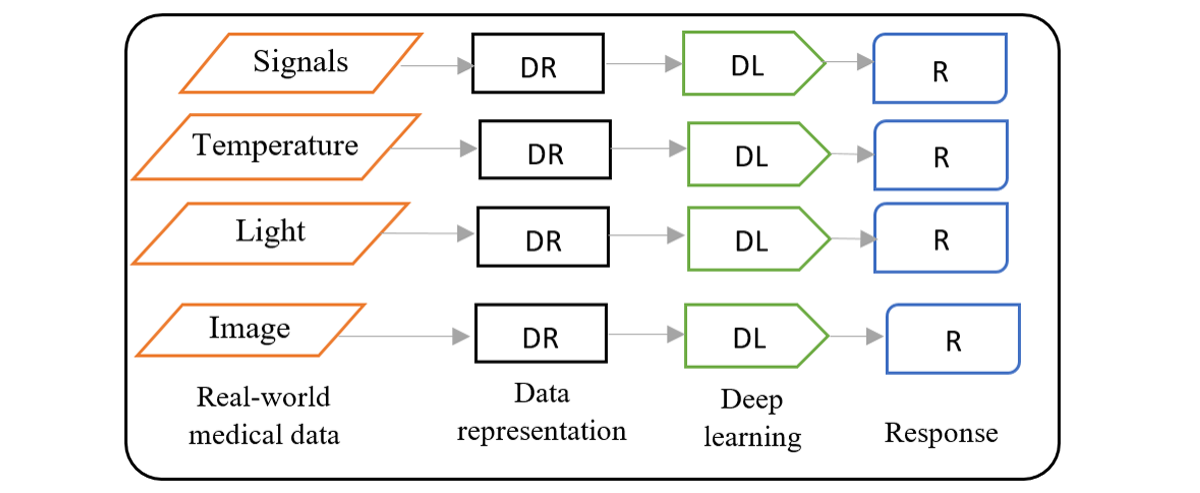
Current working technique for application of deep learning with biomedical data.

**Figure 16:**
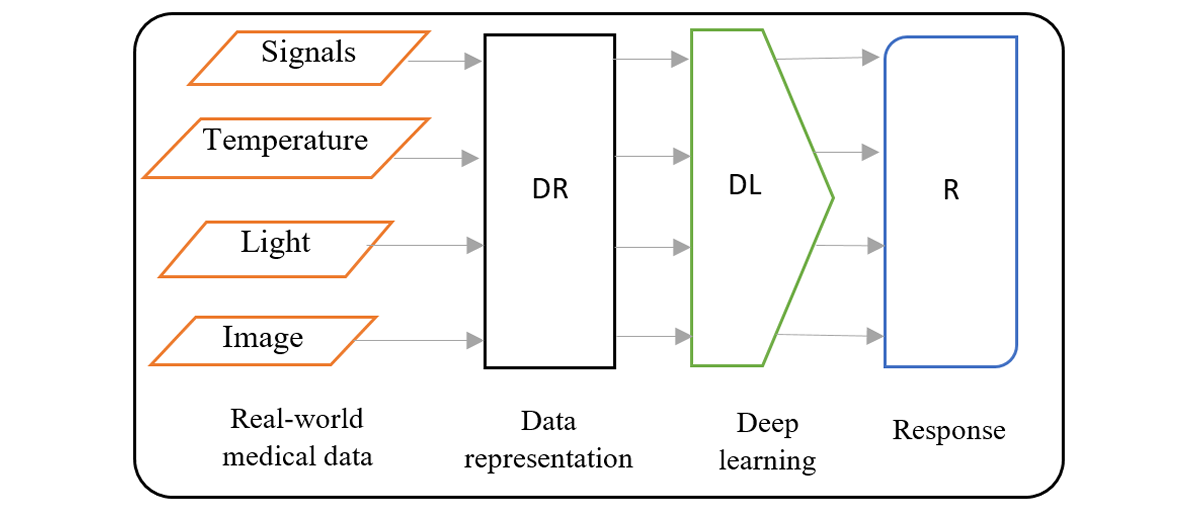
Expected required technique in biomedical application of deep learning.

Figures 15 and 16 show the differences between currently available implementation of DL methods and the expected real-world implementation, respectively. In Figure 15, different medical data have corresponding DR formats which fit a particular DL architecture. The design is expected to respond to one type of input or DR and produce a response for the expected input. However, Figure 16 is a smart DL that mimics the simultaneous process of the human body where it is capable of handling multiple inputs simultaneously to decide a response.

Consider, for example, a multiple DR DL model for monitoring a baby’s health condition. When the model observes an abnormal behavioral pattern of the baby, the temperature and the physiological signals from the baby are analyzed and considering the input of light intensity and the temperature of the environment and the baby, the model produces a response for the baby’s health status.

### Handling Biomedical Data Stream

Another challenge with DL is dealing with fast moving and streaming data [150]. There is a rapid change in the health care industry with huge volume of health care data emanating at a rapid rate. The benefit of this is that the medical practitioners can leverage on these with the support of DL model to diagnose and deliver health care services for different pathological conditions. These data can be found in real-time biomedical signals from many sources, including blood glucose monitoring, brain activity, blood pressure and oxygen saturation level, biomedical imaging from ultrasound, electrography, MRI, in thousands of terabytes for insight into medical conditions. Unstructured data format of useful patient records in the form of clinical text contains useful patterns and genomic data describing relationship between various genetic markers, disease conditions, and mutations. Physiological sensing data from ECG and EEG are important signals that are acquired from different parts of the body. It is important for DL to be able to make meaning of large volumes of continuous input data that change with time and also take into consideration when previous data become obsolete. In [151], continuous activity learning framework for streaming videos by intricately tying together deep hybrid feature models and active learning was proposed. In another architecture, a streaming hardware accelerator was proposed for incremental feature learning with denoising AE [152,153]. Although, some DL architecture variants have tried to proffer techniques for working around this situation, there are unresolved challenges regarding effective analyses of fast moving, large-scale streaming data in terms of memory consumption, feature selection, missing data, and computational complexity.

**Figure 17:**
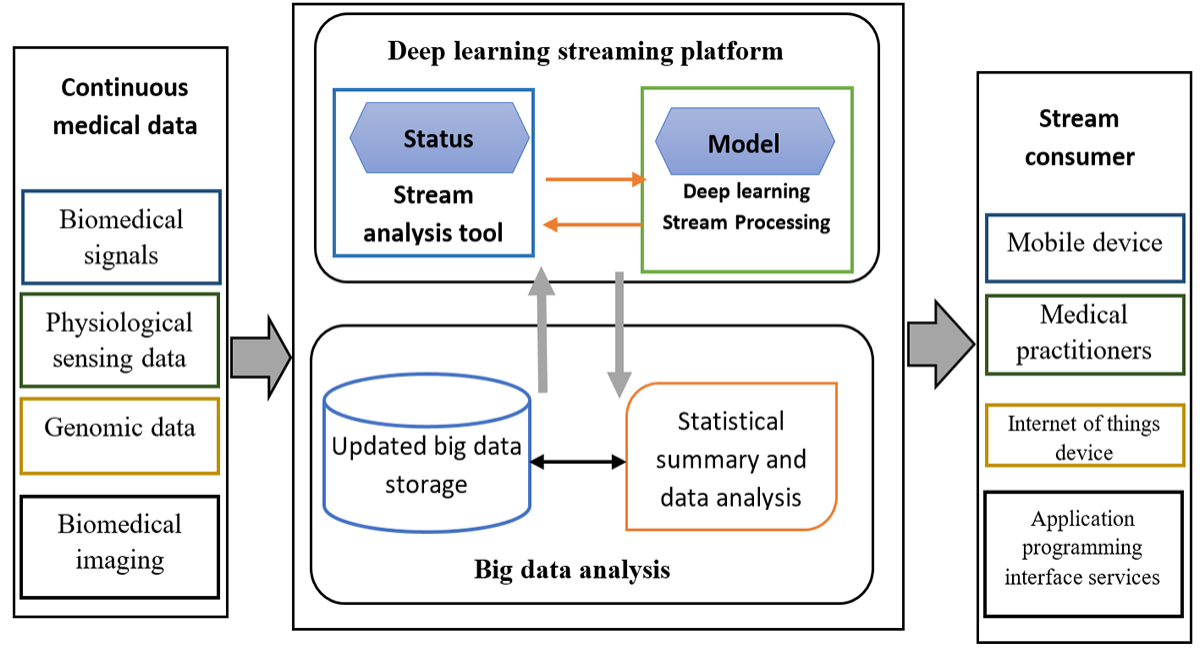
Deep learning biomedical data streaming architecture, challenges, and applications.

Figure 17 describes the summary between generated continuous medical data (CMD) and the usage of the data by DL. The challenge for DL is to make use of CMD, rather than only performing classification and prediction on the new data. The figure describes the use of updated database storage to keep generated data, which serves as a buffer to hold data until it is used to update the model in the DL stream processing.

The importance of this architecture is that it provides the platform for many consumer streams such as mobile device, medical practitioners, and third-party application programming interface (API) services to utilize the data. However, in the future, there may be variants of DL methods to handle real-time CMD, which may eliminate or reduce the function of big data analysis module.

### Analyzing Medical Big Data

Large quantity of data is responsible for highly accurate results recorded in DL. During the learning phase, features in the data are used to build or create parameters in the neurons to achieve prediction. It is important for the data to be large. In addition, it is also important for the data to contain important and required features for the training. Some medical domains that want to take advantage of DL are restricted because of the difficulty in generating or acquiring data and sometimes labeling data requires domain experts, who are not readily available. In [154], a technique was presented for dealing with fine-grained image classification scarcity. Furthermore, [155,156] presented an approach to deal with useful features in data for DL. The question is how much information is available in large data. Tuning parameters of neurons is achieved largely through validation computation procedures and the choice of DL structure. Medical big data (MBD) analysis has numerous advantages, including disease control, treatment, and diagnosis. Some MBD can be obtained from various sources, for example, medical imaging devices, the internet, biometric data, large clinical trials, biomarker data, clinical registries, and administrative claim record [157]. DL provides the tool to make smart and accurate analysis of data in this field, which will assist experts take better decision, report patient health status, and build an efficient AI. However, there are some challenges confronting DL in this domain. The major challenge is the difficulty in acquiring MBD because of danger of data misuse and lack of data sharing insensitivity which could compromise privacy of patients, legal issue, costly equipment and medical expert involved [158].

**Figure 18:**
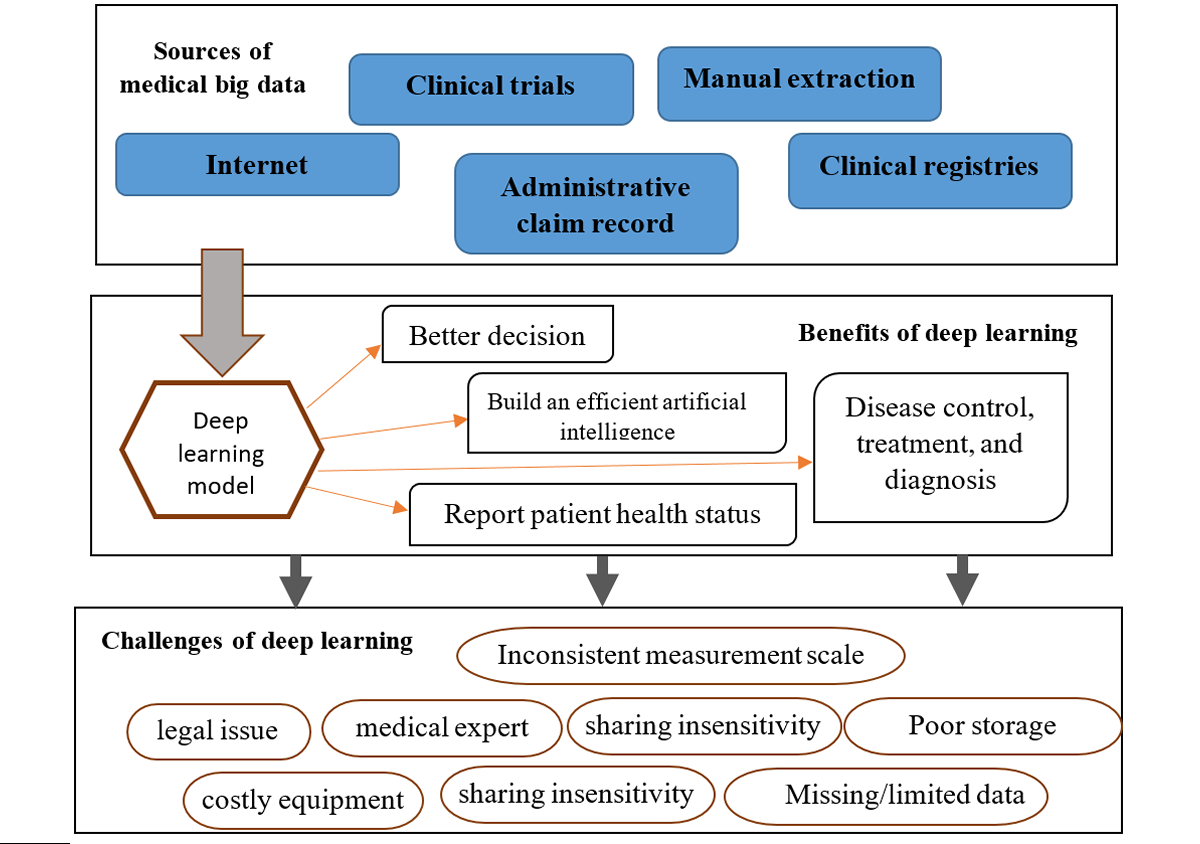
Overview of external challenges in acquiring and analyzing medical big data.

Another issue is the method of data collection which is done through application forms and protocols which could be hectic. The data are sometimes relatively small compared with data from other environments (eg, social media) and they are generated from nonreplicable situations or not readily common conditions. There are other inherent challenges encountered in MBD. Apart from missing data, there is the issue of errors in encoding medical record data during storage and difference in measurement equipment and measurement scale. In some areas, data are not available or are not enough because of the lack of knowledge on the importance of big data and data analysis. Synthetic data are sometimes constructed and integrated with acquired real data to achieve a large data size and maintain a balance of variables, nevertheless, how much trust should be given to synthetic data. In the aspect of analysis, there are different types of patient characteristics which can result in differences in physiological signals such as weight and the time of treatment, which may be an additional dimension [159]. These issues need to be resolved to provide an integrated health care system that will improve service delivery and reduce dependence on experts. DL methods require reliable and large data for successful results. Although it is relatively easy to acquire data from other sources, such as Web or user internet pattern, online customer reviews, electronic devices, and atmospheric conditions (eg, temperature and wind speed), it is often difficult to acquire data from human subjects because of the inherent challenges such as maintaining fixed position sometimes over a long period of time, static charges interference during data acquisition from the subject, difference in metabolic conditions, and negative side effects and reaction by the subject. A summary of the source of MBD, benefits of DL, and challenges of MBD is presented in Figure 18. It is necessary to have either single or multiple repositories to manage MBD; however, there are challenges such as poor network connection, incomplete or inconsistent data, and unavailable computing resources that will mitigate against achieving the desired analysis of the data.

An example is inconsistency in the measurement of date of birth. In one location the order is day, month, and year, but in another location the arrangement can be month, day, and year. In addition, there could be inconsistent units of measurement such as mmol/dl and mg/l for blood glucose measurement and ounce and kilogram for weight measurement. Although the issue of inconsistency in measurement can be managed, security and privacy still make it difficult to acquire data for analysis. Some medical institutions do not support the use of data from patients for studies or the data are not readily available. In some cases, it is expensive because of the high monetary cost needed to get this information from these institutions.

### Hardware Requirements for Medical Big Data

DL solution requires large training data to function effectively. Usually real-world medical data are very large and are constantly increasing. To implement tasks and create models, the computing machine needs to be equipped with sufficient processing power. To handle such requirements, data scientists and engineers developed multicore high performing GPUs and similar processing units as the regular central processing unit is impractical to handle large-scale DL tasks [160]. These GPUs are expensive, consume lot of power, and are not readily available for common use or in medical institutes and hospitals where the data are captured and generated. However, some companies, such as Wolfram Mathematica and Nervana Systems, have taken up the project to provide cloud-based services that allow researchers to speed up the training process [42]. The challenge is that industry level DL systems use high-end data centers, which are not available in medical institutions, whereas, deployment after training is done on smart devices, such as laptops, smart wearable devices, embedded computers, and other mobile devices, which have small and inefficient processing units. The larger the DL architecture, the bigger the computing requirement needed to accomplish training. Deploying DL solution to the real world thus becomes a costly and processor consuming situation. mHealth big data are pointless without suitable DL analytic methods to extract meaningful information and hidden patterns from data. One study [161] presents a tutorial on DL in mobile big data analytics and discusses a scalable distributed DL framework over Apache Spark. The framework is executed as an iterative MapReduce computing on many Spark workers. A partial deep model is learned by each Spark worker on a partition of the overall mobile, and a master deep model is then built by averaging the parameters of all partial models. Moreover, there are current hardware designs intended to implement artificial neurons in a chip, such as Intel Curie, NuPIC, SpiNNaker, and IBM TrueNorth [42]. There are couple of notable software packages that provide implementation of DNN and have API for Python, Java, C++, and MATLAB, for example, TensorFlow by Google, neon by Nervana Systems, and Caffe by Berkeley Center.

## Future Trends for Deep Learning

The current achievement in DL will open up more research areas and improvements on existing models. This section describes possible directions for research and development with focus on health care and applications of physiological signals.

### Complexity of Computation

There is increasing application of DL in the medical field, especially in the area of physiological signals such as ECG, EEG, electromyography (EMG), and so on. ECG measures the bioelectrical activity of the heart, EEG monitors the bioelectrical activities of the brain, and EMG observes the working condition of the muscles and nerves of the body. The success recorded in this area will bring to the surface more application and implementation variation techniques. The purpose of automated analysis of these signals is for implementation in clinical devices as a practical medical diagnostic tool to improve the efficiency of treatment and continuous health monitoring. To achieve this feat, subsequent studies need to enhance the complexity of classifier algorithm to improve computational efficiency and complexity. For example, one study showed that the memory and complexity of DBN model is higher than other algorithms such as SVM, logistic regression, and K-nearest neighbor (KNN) [139,162]. However, DBN provides high accuracy over the other algorithms. Therefore, improvement is needed to enhance the DL algorithm for practical use.

### Multitasking Deep Learning

Currently, the rise in the use of wearable devices in recent years means that multiple physiological signals can be captured simultaneously and continuously. The classification and analysis of these signals may require different DL methods for different tasks. Future studies should consider a single generalized DL method that could satisfy multiple classifications. The purpose of this approach will conserve time and effort that would otherwise be needed to create a specific method for each classification. One study [163] considered a complex scenario of learning over multivariate and relational time series with missing data, where relations are modeled by a graph. They not only predicted future values for the time series, but also to fill in missing values. Another future consideration is to design DL methods that combine multiple physiological signals for classification. Multiple signals from wearable devices provide the possibility to integrate these signals to have a unified model. Constructive application of these signals will definitely increase accuracy and will also serve multiple purposes, where when one of the signals is not available, the system can still function with available signal input. Furthermore, a combinational adaptive DL model that is capable of handling multiple physiological signals for different classification tasks (multitasking) will minimize dependence on a single model and open the opportunity for a different approach to the application of DL.

### Medical Internet of Things and Application

**Figure 19:**
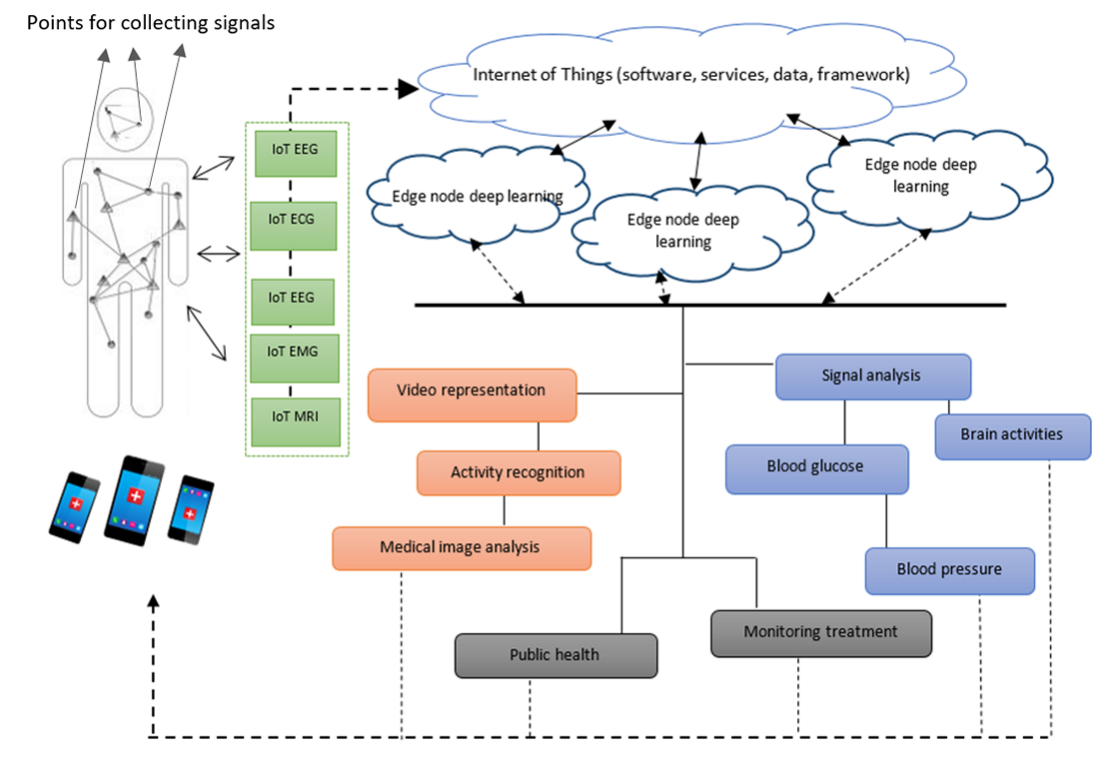
Deep learning service for medical internet of things (IoT) with edge computing and mobile apps for continuous health care monitoring using magnetic resonance images (MRI) and signals such as electrocardiogram (ECG), electroencephalogram (EEG), electromyography (EMG).

Internet of Things (IoT) and big data are responsible for the creation of smarter environment. One study [164] describes a smart environment as a physical world that is richly and invisibly interwoven with sensors, actuators, displays, and computational elements, embedded seamlessly in the everyday objects of our lives, and connected through a continuous network and smart mobility. IoT devices are on the increase; an estimated 50 billion devices will be connected to the internet by 2020 [165]. This will bring about explosion in the size of data that will be generated. The exponential growth of data from connected devices, such as wireless body sensor, smart meters, and so on, makes DL the desired tool to make meaning from these data. A cloud-based DL becomes a challenge because of connection bottlenecks and overall reduction in the quality of service owing to latency issues [166]. Edge computing is proposed to move computing service from centralized cloud servers to edge nodes closer to the end users [167]. Some research has been conducted in this emerging field, such as seizure prediction in controlling epilepsy in medically refractory patients with EEG and electrocorticography signals via IoT [168]. The rapid proliferation of mobile phone and wearable devices has contributed to the evolution of IoT-enabled technology from usual single center–based system to more personalized health care systems. mHealth uses the wireless connection in IoT and mobile technology from mobile industry to create a connection between patients and health care professionals to make patients become advocates of their own health and promote communication between the professional and patients. mHealth framework in IoT has been used to create a voice pathology detection system using DL [169]. In the system, voices are captured using smart mobile devices. Voice signals are processed before being fed to a CNN. One study [170] presented a DL and mHealth technologies for improving tuberculosis diagnosis in Peru. Figure 19 shows the structure of edge computing technology where all the data captured from IoT devices are stored in the cloud. The edge nodes use specific data from the cloud required by client devices to perform DL analysis and computation. Edge computing adds 2 major enhancements to cloud computing by processing large volumes of data before transferring it to the cloud and enabling computing ability in the edge node, which optimizes the resources in the cloud. An example is DL-based food recognition system for dietary assessment on an edge computing service infrastructure [171]. Further research can be done to improve this area for efficient implementation of DL, for example, a distributed, layer-centered DL architecture that supports edge node operation of cloud resources. DL techniques maximize the number of tasks in computing environment owing to limited service capability, network performance, and scalability.

Performance evaluation and measurement of DL on edge computing is also another area to consider. Moreover, in the future there will be distributed and integrated variants of DL methods for edge computing because of the increase in IoT devices and technology.

### Semisupervised Learning for Biomedical Data

Another key area of interest would be to explore the situation of both labeled and unlabeled data which occur in many biological domains such as proteins and DNA. The objective of DL in such cases is to integrate semisupervised training techniques toward achieving the criteria for good DR learning. For further studies to enable DL understand the patterns and DRs in such situations (unlabeled/unsupervised data), one approach would be to consider the existing labeled/supervised data to tune learned pattern and representation to achieve the optimal modeling for the data. Another approach is to combine DL and active learning [151]. Variants of semisupervised learning in data mining and active learning methods such as adaptive learning and transfer learning can be exploited toward obtaining improved DRs. Input from crowdsourcing or human experts can be used to obtain labels for some data samples which can then be used to better tune and improve the learned DRs [150]. Furthermore, hybrid DL has been constructed for feature extraction, classification, and verification of faces [172]. Application of DL in physiological signals for health care monitoring and analysis is still in its infancy and often suffers from incomplete or unavailable labeled data. More study needs to be done in this area and the implementation of hybrid techniques and variants of semisupervised learning to overcome the challenges of unlabeled data.

### Replacement of Biomedical Research Methods by Deep Learning

There are more areas to implement DL to improve services, operations, devices, and software for health and medical fields. One study [173] proposed a clinical validation technique for improving grading of data collected by crowdsourcing for diabetic retinopathy, a leading cause of vision loss because of diabetes mellitus. A logistic regression method was implemented with 50% dataset for training that have normal and abnormal classification labels. Test and validation was performed on 50% dataset. The result achieved 90% sensitivity. However, the operation requires human decision which is prone to error and bias. CNN can be applied to 50% of labeled images to learn the features through series of convolution and pulling layers to predict 50% test set. The sensitivity is expected to be more than 90% as CNN abstraction of features at different layers will improve the sensitivity results. Furthermore, analysis of fall of individuals with dementia from continuous video monitoring was performed for early detection and prevention [174]. Analysis was carried out using a 4-point Hopkins Falls Grading Scale. A suitable DL method will be a hybrid technique of both RNN and CNN (recurrent CNN), which is able to approximate a function from a series of video frames in the continuous video sequence to systematically determine patient’s condition: prefall, fall, and postfall. This can be implemented to trigger alarm for medical attention for the patient. In [175], mainstream wearable device was presented in health monitoring to support consumers in making purchasing decision. The analysis method implemented may become ineffective as the data grows larger, but a DNN will remain effective despite the size of the data and the performance will not decline. Another area of application is in cardiac auscultation that can provide information about cardiovascular hemodynamics and diseases with simple diagnostic algorithm [176]. LSTM technique will effectively map the sequence of sounds from the device capturing the sound to distinguish between normal and pathologic heart sounds. LSTM is capable of understanding the pattern from the sound data because of the gate and memory circuit which is an integral part of the DL algorithm. One study [177] presented a qualitative and quantitative tablet-based software application for assessing bodily symptoms for both clinical and research purposes. The implementation can be achieved with multilayer stack AE between patients and doctors. The architecture of AE is able to encode and decode the input from patients to the expected output for the treating doctor and vice versa. The multilayer concept will handle the test-retest reliability. Furthermore, a construction of a priori analysis was employed to describe the essential qualities of participant’s experience [178]. This included delineation of common and novel themes relating to informed consent, with a self-administered, mobile phone-based electronic consent (eConsent) process over a 6-month period within the Parkinson mPower app. This challenge can effectively be resolved with structured DBN architecture. Data collected for the specified period can be used to train a DBN model that resides in a cloud and the mPower app can communicate with the model to get required result. DBN layer-wise training technique ensures that features in the input data are taken into consideration during the creation of the model. This architecture removes processing responsibility from the mobile app (this makes the device light), provides the possibility for extension of the model, allows multiple users to take advantage of the system, and allows for central update when necessary. In [179], user needs for mobile behavioral-sensing technology was presented for depression management using thematic analysis with an inductive approach. The research was conducted by interviewing 9 clinicians and 12 students with depression, recruited from a counseling center. The interview duration was between 40 and 50 min and there was audio recording and transcription. The success recorded was because of small data size. However, it will become challenging with large size of data and human limitation will affect performance. Therefore, hybrid DL technique will provide a better performance, with the use of LSTM to model the recorded audio and DNN for the structured content. The use of hybrid technique will make better meaning from the data, as the model keeps learning and improving with increasing available data without human bias or the limitation of thematic analytic model. DL can be implemented to understand the use of gyroscope for classification of physical activities using mobile phone motion sensor. In [180], 13 physical activities were considered, and the classification technique required the use of many algorithms: C4.5, Naive Bayes, logistic regression, KNN, and meta-algorithms such as boosting and bagging. DL technique called RBM will be appropriate to replace the implemented algorithms. The conditional distribution over the hidden nodes in RBM makes the feature presentation of each activity from the input signal possible.

### Conclusions

ML is gradually influencing the way health care treatment and monitoring is performed. All of this can be attributed to the success recorded by DL. Compared with conventional ML and feature engineering, DL has potentially proven to provide response to data analysis and learning problems found in enormous volumes of data. Different variations of DL techniques have been implemented across many areas such as biomedical image, health record processing, sensors and physiological signal processing, human motion and emotion analysis, and so on. A successful AI system must have an excellent ML component; DL is taking the position as the number one choice for AI. To understand DL, in this review paper, we discussed about the basic architecture of DL methods. The discussion focused on principles of operation and application in health and medical domains. We presented the following models: (1) AE, (2) RNN, (3) CNN, (4) DBN, and (5) DBM. We presented a review of publications that have implemented these models in medical image, physiological signals, biological system, and EHR. We investigated the trend of DL implementation from 2012 to 2017. We observed a steady rise, with CNN having the highest increase occurrence. Computer and network architecture will gradually begin to change to support big data and DL techniques for efficiency and scalability. Moreover, there are some inherent challenges encountered in DL that need to be addressed. Most of these data in the real world are in unstructured format that cannot be processed by DL methods and require extra layer of encoding and representation. Clinical data are expensive to acquire and dataset contains incomplete and inconsistent records.

Statistical results presented in this review paper reveals that future applications and trends in DL will see more application of CNN implemented in medical image processing. There will be more variations of DL techniques across the general DL methods. There will be increase in the application of physiological signals using DL methods for diagnosis. The advancement in IoT and edge computing technology will bring about a different model of DL that will support this technology. Further research and study need to consider targeting this platform and solve issues relating to performance evaluation, scalability, and limited service capability. AI for mHealth will be driven by DL assisted by cloud and edge computing to process big data from wearable and mobile devices. Therefore, we can say that DL offers an excellent algorithm and is the answer to the challenges presented by MBD. However, the use of DL in every application that requires data analysis should not be done at the expense of other ML algorithms with less computation and memory requirement that are capable of producing similar results. Furthermore, attention should be given to other ML algorithms that have good possibility of achieving high performance with big data to deal with the demand for data analysis.

## Abbreviations

2D: two-dimensional
3D: three-dimensional
AE: autoencoder
AI: artificial intelligence
API: application programming interface
BM: Boltzmann machine
CMD: continuous medical data
CNN: convolutional neural network
CRF: conditional random fields
CT: computed tomography
DBM: deep Boltzmann machine
DBN: deep belief network
DBN-GCs: deep belief network with glia chains
DL: deep learning
DNN: deep neural network
DR: data representation
ECG: electrocardiogram
EEG: electroencephalogram
EHR: electronic health record
EMG: electromyography
FC: fully connected
GPU: graphics processing unit
IoT: Internet of Things
KNN: K-nearest neighbor
LSTM: long short-term memory
MBD: medical big data
mHealth: mobile health
ML: machine learning
PPG: photoplethysmogram
RBM: restricted Boltzmann machine
ReLU: rectified linear unit
RNN: recurrent neural network
scRNA-seq: small cytoplasmic RNA sequence
SSAE: stacked sparse autoencoder
SVM: support vector machine
